# PD-L1-targeted OMV priming licenses myeloid co-stimulation and chemokine circuits to potentiate CAR-T therapy in advanced-stage solid tumors

**DOI:** 10.1186/s12951-026-04722-6

**Published:** 2026-06-23

**Authors:** Yueyao Yang, Junjie Peng, Jing Wei, Jia Qin, Yang Jiao, Ming Liu, Gang Wang

**Affiliations:** 1 National Engineering Research Center for Biomaterials, College of Biomedical Engineering, Sichuan University, Chengdu, 610064 Sichuan Province China; 2 Department of Medical Oncology, Gastric Cancer Center, West China Hospital, Sichuan University, Chengdu, 610041 Sichuan Province China

**Keywords:** CAR-T, Bacterial outer membrane vesicles, PD-L1, Ttumor microenvironment, Single-cell RNA sequencing, Dendritic cells, Macrophage reprogramming, Co-stimulation

## Abstract

**Background:**

Chimeric antigen receptor T (CAR-T) cell therapy has achieved remarkable success in hematologic malignancies, yet its efficacy in solid tumors is frequently limited by an immunosuppressive tumor microenvironment (TME) that restrains trafficking, persistence, and effector function. Targeted priming strategies that remodel myeloid compartments and reinforce intratumoral co-stimulation may help overcome these barriers.

**Methods:**

We engineered bacterial outer membrane vesicles displaying PD-L1-targeting single-chain variable fragment (OMV^PD−L1 scFv^). Their tumor-targeting and immunomodulatory functions, including DC maturation, co-stimulatory capacity, macrophage polarization, and phagocytosis, were evaluated in vitro. The potent antitumor efficacy and TME remodeling were examined in syngeneic models, specifically highlighting its synergy with dose-de-escalated CAR-T therapy. Single-cell RNA sequencing (scRNA-seq) further elucidated the mechanisms of early immune rewiring and intercellular communication programs associated with OMV priming.

**Results:**

OMV^PD−L1 scFv^ demonstrated enhanced tumor accumulation following systemic administration and promoted myeloid licensing by increasing DC maturation and co-stimulatory competence while repolarizing macrophages toward pro-inflammatory, tumor-phagocytic program. In vivo, OMV^PD−L1 scFv^ remodeled the TME and improved CAR-T activation, tumor trafficking, and antitumor efficacy, achieving robust tumor control even at reduced CAR-T dosages in advanced solid tumors. scRNA-seq analyses highlighted transcriptomic signatures consistent with coordinated lymphoid and myeloid remodeling, characterized by enriched profiles of improved T-cell functional states, a potential transcriptomic basis for *Xcl1*-mediated DC recruitment, strengthened DC-to-T co-stimulatory CD28 axis, and predicted enrichment of chemokine-driven myeloid–lymphoid communication involving *Ccl3/4/5–Ccr5*.

**Conclusions:**

PD-L1-directed OMV priming provides a modular, systemic strategy to reprogram the solid-tumor immune ecosystem by licensing myeloid compartments and reinforcing intratumoral co-stimulation, thereby supporting improved CAR-T recruitment and function. These findings establish engineered OMVs as a mechanistically grounded priming platform to complement CAR design for solid tumor immunotherapy.

**Graphical abstract:**

**Figure:**
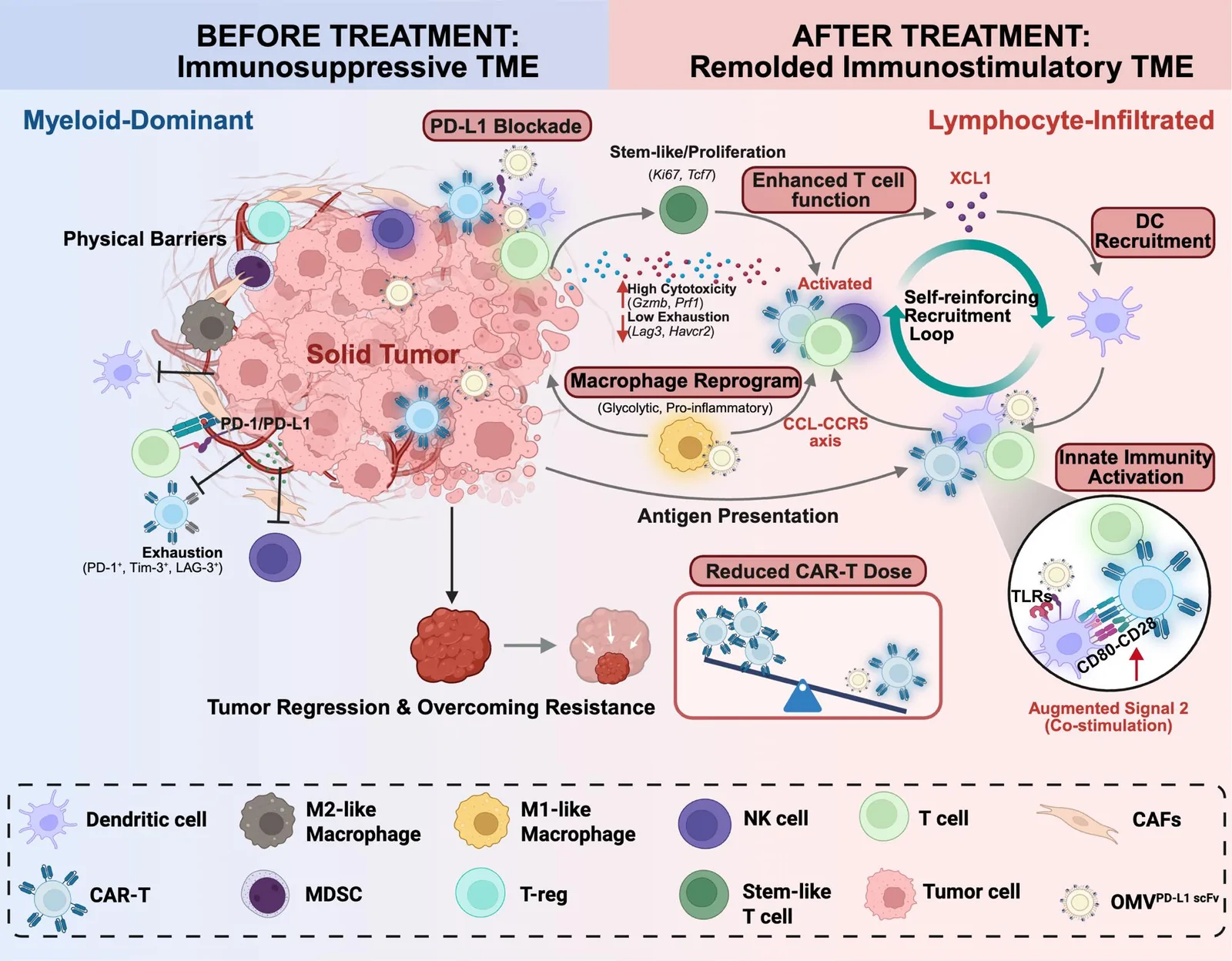

**Supplementary Information:**

The online version contains supplementary material available at https://doi.org/10.1186/s12951-026-04722-6.

## Background

 Chimeric antigen receptor T (CAR-T) cell therapy has transformed the treatment landscape for B-cell malignancies [1–3]. However, translating this success to solid tumors remains a major challenge [4–6]. In solid cancers, limited efficacy is frequently driven not only by the lack of tumor antigens per se, but also by a hostile tumor microenvironment (TME) that constrains CAR-T cell trafficking, persistence, and effector function [4, 5, 7–10]. Multiple layers of suppression collectively restrict effector lymphocyte accumulation and promote dysfunctional states after tumor entry, including insufficient antigen presentation, myeloid-dominant inhibitory niches, checkpoint signaling, metabolic constraints, and physical barriers [10–14]. Consequently, even CAR-T cells equipped with potent intracellular co-stimulatory domains can exhibit attenuated activity in vivo when the surrounding tissue context fails to support sustained activation and cytotoxicity, including through vesicle-mediated dysfunction and extracellular matrix constraints [9, 10, 15, 16].

Among immunoregulatory pathways shaping the TME, the PD-1/PD-L1 axis is a central mechanism of immune evasion [17–20]. PD-L1 is broadly expressed by malignant cells and tumor-associated myeloid cells, including macrophages and dendritic cells (DCs), where it can dampen T-cell activation at the immunological synapse [12, 18, 20]. Effective T-cell responses in tumors require antigen recognition, as well as adequate co-stimulatory signaling through CD28 [21, 22]. Tumor-associated antigen-presenting cells often display impaired maturation and reduced functional co-stimulation, creating a context in which checkpoint blockade alone may be insufficient to restore antitumor immunity fully [12, 23]. Strategies that simultaneously engage PD-L1-mediated suppression and enhance antigen-presenting cell licensing could therefore be particularly valuable for adoptive cell therapies, which depend on both efficient tumor homing and a supportive intratumoral niche [21–24].

A variety of approaches have been explored to overcome the barriers imposed by the solid-tumor TME. These include checkpoint blockade combinations [17], cytokine/chemokine-armored CAR-T designs and chemokine receptor engineering [25, 26], innate activation strategies and immunocytokines [23, 24], oncolytic platforms [27], and engineered nanomedicines [28, 29]. Despite encouraging activity in selected settings, many strategies remain constrained by incomplete tumor localization and heterogeneous responses [4–6, 28]. More fundamentally, they often fail to establish a mechanistic link between innate immune activation and durable effector lymphocyte function [22–24]. An effective priming approach for CAR-T therapy should therefore be systemically administrable and manufacturable, while preferentially enriching immunomodulatory activity within tumors [28, 29]. Such a strategy would reprogram suppressive myeloid compartments into immunostimulatory states and couple innate activation to sustained lymphocyte recruitment and co-stimulation [13, 14, 21–24].

Bacterial outer membrane vesicles (OMVs) provide an attractive platform to meet these criteria [30–32]. OMVs are nanoscale lipid bilayer vesicles naturally released from Gram-negative bacteria and enriched in microbe-associated molecular patterns that can activate innate immune pathways [30]. They have been investigated as vaccine adjuvants and delivery vehicles, owing to their modularity and amenability to surface engineering [31, 32]. Notably, OMV-based platforms can engage antigen-presenting cells, promote inflammatory signaling, and reshape local immune landscapes—features that make them well-suited as immune “primers” before cellular immunotherapy [31–35]. However, native OMVs lack tumor specificity, motivating rational engineering strategies that spatially focus their immune-modulatory effects within tumors [33, 35, 36].

PD-L1 provides a biologically rational and clinically relevant handle for tumor-directed immune priming [18–20]. PD-L1 expression is frequently elevated in many solid tumors and is also induced on intratumoral myeloid cells during inflammation [12, 18, 20]. Targeting PD-L1 may therefore provide a rational handle to localize immunomodulatory agents within PD-L1–positive tumor and stromal compartments, while simultaneously engaging checkpoint biology at key sites of immune regulation [18–20]. Moreover, because productive T-cell activation requires CD28 co-stimulation through CD80 and CD86, approaches that promote myeloid maturation and reinforce CD80/CD86–CD28 signaling offer a mechanistic bridge between innate activation and improved effector function of both endogenous T cells and adoptively transferred CAR-T cells [21–24].

Here, we engineered outer membrane vesicles displaying a PD-L1-targeting single-chain variable fragment (OMV^PD-L1 scFv^) to integrate tumor-directed PD-L1 engagement with OMV-intrinsic innate immune activation as a priming strategy for CAR-T therapy in advanced-stage solid tumors. Using complementary in vitro assays, syngeneic tumor models, and single-cell transcriptomic profiling, we show that OMV^PD-L1 scFv^ reshapes the tumor immune ecosystem. It licenses myeloid compartments, strengthens intratumoral co-stimulation through the CD80–CD28 axes, and rewires myeloid–lymphoid communication to support lymphocyte recruitment and function. OMV^PD-L1 scFv^ priming overcomes physical and immunosuppressive barriers, facilitating superior CAR-T infiltration and sustained antitumor activity.

## Methods

### OMV production and characterization

To generate engineered OMVs displaying the PD-L1-targeting moiety, the DNA sequence encoding the anti-PD-L1 scFv (derived from atezolizumab) was fused in-frame to the C-terminus of the *E. coli* outer-membrane anchor protein ClyA, followed by a 6×His tag. This ClyA-PD-L1-scFv-6×His expression cassette was cloned into the pET28a vector. Similarly, to generate the isotype control OMV^Her2−scFv^, an identical expression cassette was constructed by substituting the anti-PD-L1 scFv with an anti-HER2 scFv derived from trastuzumab. The recombinant plasmids were then transformed into *Escherichia coli* Rosetta (DE3) competent cells. A single positive colony was inoculated and cultured at 37 °C in LB medium containing kanamycin (50 µg/mL; 1162GR005, Biofroxx) and chloramphenicol (34 µg/mL; ST1150, Beyotime) with shaking (220 rpm) overnight. The following day, the overnight culture was subcultured at a 1:200 ratio into fresh LB medium containing the same antibiotics and grown at 37 °C with shaking (220 rpm) for approximately 6 h until the OD600 reached 0.7. Protein expression was then induced by adding a 0.1 M IPTG (ST098, Beyotime) stock solution to a final concentration of 0.6 mM. The induced culture was subsequently incubated at 16 °C with shaking at 180 rpm for 20 h. All subsequent isolation, purification, and characterization procedures were applied identically to both OMV^PD−L1−scFv^ and OMV^Her2−scFv^.

Bacteria were removed by centrifugation (4 °C, 8,000 rpm, 30 min), and supernatants were passed through a 0.45 μm vacuum filter (41112, Labselect). Clarified supernatants were concentrated using a 100 kDa ultrafiltration device (Sartorius) and then sterilized by filtration through a 0.22 μm PES membrane (SLGPR33RS, Millipore). The concentrated filtrate was ultracentrifuged at 150,000 g for 3 h at 4 °C to obtain OMVs (Optima MAX-XP, Beckman). The precipitate was washed once by centrifugation again with PBS. OMVs were further purified using Exosome purification column (ES911, Echo Biotech) and stored at -80 °C pending further use. Total OMV protein was quantified using a BCA protein assay kit (23227, Thermo Scientific). Hydrodynamic size distributions were measured by dynamic light scattering (Zetasizer Nano ZS90, Malvern). Vesicle morphology was assessed by transmission electron microscopy (Tecnai G2 F20 S-TWIN, FEI).

### Cell culture

CT26, and CT26-mCD19-Luc (CT26 cells transduced to express mouse CD19 and luciferase) were cultured in RPMI 1640 (Gibco) supplemented with 10% fetal bovine serum (FBS; Gibco) and 1% penicillin-streptomycin (15140122, Gibco). HEK293T, Plat-E, A20, A20-Luc, Hepa1-6-chGPC3 (Hepa1-6 cells transduced to express a chimeric GPC3), and CHO-mPD-L1 (CHO-K1 cells transduced to express mouse PD-L1) were cultured in DMEM (Gibco) supplemented with 10% FBS and 1% penicillin-streptomycin. The human-mouse GPC3 chimera (chGPC3) was generated by replacing Lys538-Pro558 in mouse GPC3 (GenBank: NM_016697.3) with Ser539-His559 from the GC33-binding epitope in human GPC3 (GenBank: NM_001164617.1). All cells were maintained at 37 °C with 5% CO_2_. Cells between passages 2 and 10 were used. The Plate-E cell line was purchased from Cellverse Co., Ltd (Shanghai, China). All other cell lines were obtained from National Collection of Authenticated Cell Cultures (Shanghai, China) in 2022.

### Fluorescent labeling of OMVs

For cell-binding assays, OMVs and OMV^PD−L1 scFv^ were labeled with DiI (HY-D0083, MCE) following the manufacturer’s instructions. Free dye was removed by ultracentrifugation (150,000 g, 3 h, 4 °C), and labeled vesicles were resuspended in PBS.

For in vivo biodistribution studies, OMVs and OMV^PD−L1 scFv^ were labeled with DiR (HY-D1048, MCE) for 30 min at room temperature. Free dye was removed by ultracentrifugation (150,000 g, 3 h, 4 °C), and labeled vesicles were resuspended in PBS for injection.

### Nano-Flow cytometry

Mouse PD-L1-Fc protein was labeled with an Alexa Fluor 488 labeling kit (R10709, Invitrogen). OMVs or OMV^PD−L1 scFv^ (100 µL; 100 µg/mL) were incubated with 1 µg of labeled PD-L1-Fc at 4 °C for 60 min. Excess PD-L1-Fc was removed by two rounds of ultracentrifugation (150,000 g, 3 h, 4 °C). Vesicles were gently resuspended in PBS and analyzed on a Flow NanoAnalyzer (NanoFCM, China).

### CD28-Fc binding assay

BMDCs were treated with OMVs, OMVs plus soluble PD-L1 scFv, or OMV^PD−L1 scFv^ (10 µg/mL) for 12 h at 37 °C, washed three times with PBS, and incubated with recombinant mouse CD28 (C-Fc-6 × His) (CI67, Novoprotein) for 2 h at 4 °C. Cells were washed and stained with FITC-conjugated goat anti-human IgG (H + L) (1:20; SA00003-12, Proteintech) for 1 h at 4 °C and analyzed by flow cytometry. The PBS, OMV, and OMV^PD−L1 scFv^ (1 mg/kg) were subsequently injected intravenously at the base of the tail in mice. After 24 h, single-cell suspensions from the lymph nodes were obtained and subjected to three washes with PBS. The subsequent steps were performed following the same procedure as described above.

### Mice

C57BL/6, BALB/c and B6.SJL-Ptprc^a^ Pepc^b^/BoyCrl (B6-CD45.1) mice were procured from Charles River (Beijing, China) and maintained at the animal facility of the Sichuan University in a pathogen-free environment, with an ambient temperature of 22 °C and a humidity content of 50%. These mice were sustained on a regular diet and hydration schedule, under a cycle of 12 h of light followed by 12 h of darkness.

For the A20 lymphoma model, 2 × 10^6^ A20 cells were inoculated subcutaneously into the right flank of female BALB/c mice (6–8 weeks). For the CT26 colorectal carcinoma model, 1 × 10^6^ CT26 cells were inoculated subcutaneously into the right flank of female BALB/c mice (6–8 weeks). When tumors reached approximately 100 mm^3^, mice were allocated to groups with comparable mean tumor volumes and treated intravenously with PBS, soluble PD-L1 scFv (1 mg/kg), OMVs, OMVs plus PD-L1 scFv, or OMV^PD−L1 scFv^ (1 mg/kg; expressed as total vesicle protein) every 3 days for three doses or as a single dose where indicated.

For combination therapy in advanced tumors, 1 × 10^6^ CT26-mCD19 cells were implanted subcutaneously in BALB/c mice. When tumors reached approximately 400 mm^3^, mice received PBS or OMV^PD−L1 scFv^ (1 mg/kg, i.v.). Twenty-four hours later, mice received αmCD19-CAR-T cells (low dose: 1 × 10^6^ viable cells in 150 µL PBS; high dose: 5 × 10^6^ viable cells in 150 µL PBS, i.v.).

For the advanced hepatocellular carcinoma model, 5 × 10^6^ Hepa1-6-chGPC3 cells were implanted subcutaneously in C57BL/6 mice. When tumors reached approximately 350 mm^3^, mice received PBS or OMV^PD−L1 scFv^ (1 mg/kg, i.v.), followed 24 h later by αGPC3-CAR-T cells (1 × 10^6^ viable cells, i.v.). Blood and tumors were collected 5 days after CAR-T infusion for flow cytometry where indicated. In addition, serum was obtained 2 days after CAT-T infusion for cytokine multiplex analysis (XMPlex03240771, Sci-XM Biotechnology, Wuhan, China).

Tumor volumes were calculated as: Volume = (length × width^2^)/2. All animal experiments were approved by the Biomedical Research Ethics Committee of West China Hospital, Sichuan University, and conducted in accordance with AAALAC guidelines. Mice were euthanized if the maximal tumor diameter reached 20 mm or if humane endpoints were met (including labored breathing, abnormal movement, hypothermia, hunched posture, or > 20% body-weight loss).

### Tumor dissociation and flow cytometry

Tumors were harvested and mechanically dissociated using a shaker for 30 min at 37 °C in a digestion solution of 1 mg/mL collagenase I (SCR103, Sigma), 1 mg/mL collagenase IV (C4-BIOC, Sigma) and 100 µg/mL DNAse I (10104159001, Roche) in RPMI 1640 medium. Digested tissues were filtered through a 40 μm strainer. Red blood cells were lysed with ACK lysis buffer (5 min, room temperature). Cells were washed twice and resuspended in FACS buffer (PBS containing 5% BSA).

For staining, 5 × 10^6^ cells were incubated with a fixable viability dye (Invitrogen) and anti-CD16/32 Fc block (14-0161-82, Invitrogen) for 15 min at 4 °C, followed by antibody staining for 30 min at 4 °C. After washing, samples were fixed with IC Fixation Buffer (00-8222-49, Invitrogen) for 45 min at 4 °C. Intracellular staining (Ki67, Foxp3, Granzyme B, CD206) was performed using the Foxp3/Transcription Factor Staining Buffer Set (00552300, eBioscience) according to the manufacturer’s instructions.

Acquisition was performed on an LSRFortessa cytometer (BD Biosciences) and data were analyzed using FlowJo v10 software (Tree Star, Ashland, OR). Gating strategies were based on live singlets, followed by CD45^+^ leukocytes where applicable.

### Lymph node immune responses and flow cytometry

The animal model establishment and therapeutic dosing regimens for this experiment were identical to those used in the Hepa1-6-chGPC3 model described previously. To evaluate systemic immune activation and T-cell priming, tumor-draining lymph nodes (tdLNs) and contralateral non-draining lymph nodes (ndLNs) were harvested from mice 3 days after OMVs injection. The harvested lymph nodes were mechanically dissociated by gently grinding the tissues through 70-µm cell strainers using the plunger of a syringe in cold PBS to obtain single-cell suspensions. The suspensions were then divided into two parallel panels for DC and T-cell profiling. For DC analysis, cells were pre-incubated with anti-CD16/CD32 (Fc block) and surface-stained with fluorophore-conjugated antibodies against CD45, F4/80, CD11c, MHCII, CD80, CD86, CD103, and CCR7. For the detection of tumor-antigen-specific T cells, a Mouse tumor antigen-specific T cell detection kit (ESMDT-HEPA16-1, ES-BIO) was utilized. Briefly, cells (2 × 10^6^ cells/mL) were co-incubated with detection nanoparticles (1.0 mg/mL) for 40 h, with Brefeldin A added during the final 4 h. The harvested cells were stained with a viability dye, Fc-blocked, and surface-stained for CD3, CD4, CD8, CD44 and CD62L. Subsequently, the cells were fixed with IC Fixation Buffer, permeabilized, and stained intracellularly for IFN-γ. All prepared samples were acquired on a flow cytometer, and the data were analyzed using FlowJo software.

### scRNA-seq analysis

Data analysis was performed using R (v.4.5.2) and Seurat (v.5.4.0). Cells were retained only if they met the following criteria: the number of detected genes (nFeature_RNA) was between 500 and 5,000; the total number of unique molecular identifiers (UMI counts/nCount_RNA) ranged from 1,000 to 50,000; the percentage of mitochondrial gene expression (percent.mt) was less than 10%. Doublets in each sample were predicted and excluded using ‘DoubletFinder’ (v.2.0.6). Filtered gene-barcode matrices were merged and normalized using the ‘NormalizeData’ function. Highly variable features were selected using the ‘FindVariableFeatures’ function, typically focusing on the top 2,000 features. Then, data were scaled using the ‘ScaleData’ function with ‘vars.to.regress’ parameter set to ‘S.Score, G2M.Score’ to regress out the influence of cell cycle. Subsequently, Harmony batch correction was applied to rectify PCA embeddings to mitigate technical batch effects across experiments.

The ‘RunPCA’ function was used to perform principal components analysis and the top 50 principal components were subjected to perform uniform manifold approximation and projection (UMAP) and cell clustering (resolution = 0.1). Differentially expressed genes (DEGs) were identified using the ‘FindAllMarkers’ function, applying a Wilcoxon Rank-Sum test with a log2 fold-change threshold of 0.25. The expression level of cluster-specific and cell-type marker genes was visualized using dot plots, violin plots, and feature plots. The ‘AddModuleScore’ function in Seurat package was used to quantify the activity of specific gene sets within individual cells. CellChat (version 1.6.1) to infer the cell-cell interaction network. The communication probability between two cell subsets was predicted based on the expression of ligand-receptor pairs using default parameters. For in-depth analysis of cell subtypes, T cell, DCs and macrophage were extracted for new PCA embeddings, nearest-neighbor graphs and harmony batch corrections. The paired quantile–quantile plot was calculated using the Wilcoxon rank-sum two-sided test between the two treatment conditions.

### Statistical analysis

Statistical analyses were conducted using GraphPad Prism version 9.4.1 (GraphPad Software, USA). Comparison between two groups, Student’s t-test was applied, while for multiple groups, a one-way ANOVA followed by Tukey’s multiple-comparisons test was used. Survival analyses were performed using the Kaplan-Meier method with significance evaluated through the log-rank test. n values denote independent biological replicates. Data are presented as Mean ± SEM unless specified. Differences were considered significant if *P* < 0.05. There is an estimate of variation within each group, and the variance between groups is similar. All of the results from the study were included for statistical analysis with no blinding involved. No data points were excluded.

## Results

### Engineering and characterization of PD-L1-directed OMVs

To generate OMVs displaying an anti-PD-L1 single-chain variable fragment (scFv), we derived the scFv sequence from atezolizumab and fused it to the *E. coli* outer-membrane anchor ClyA with a C-terminal 6× His tag. *E. coli* Rosetta (DE3) carrying the ClyA-scFv expression plasmid was induced with IPTG, and OMVs were isolated from the culture supernatant by ultrafiltration and centrifugation (Supplementary Fig. S1). We refer to these engineered vesicles as OMV^PD−L1 scFv^.

**Fig. 1:**
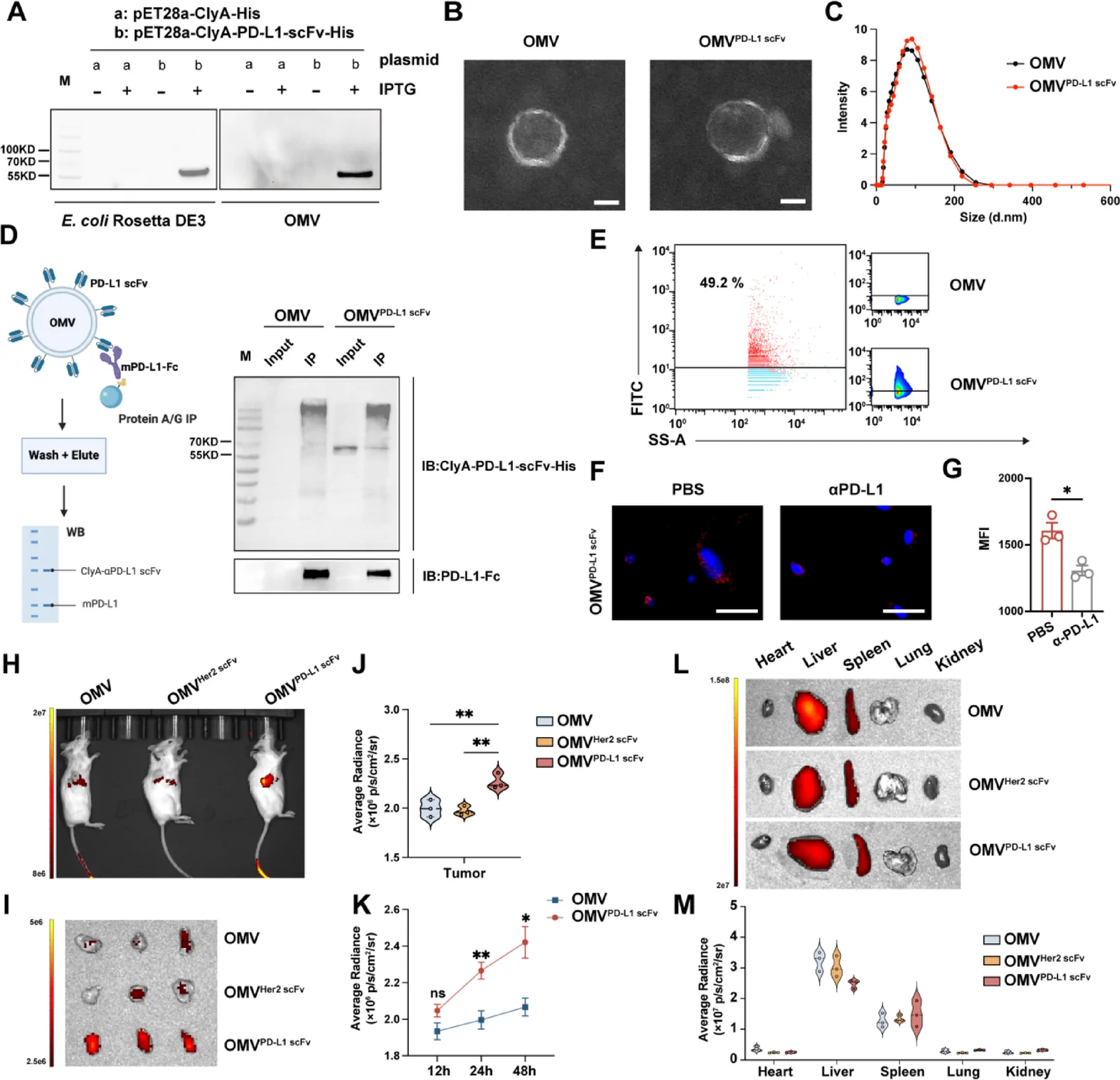
Engineering, characterization and tumor targeting of PD-L1-directed OMVs. (**A**) Immunoblot analysis of ClyA–PD-L1 scFv–6×His expression in *E. coli* Rosetta (DE3) lysates and purified vesicles, detected with anti-His antibody; IPTG was used for induction. (**B**) Transmission electron microscopy images of parental OMVs and OMV^PD−L1 scFv^. Scale bars: 20 nm. (**C**) Hydrodynamic diameter distributions measured by dynamic light scattering. (**D**) Co-immunoprecipitation (IP) schematic (left) and immunoblotting (right) showing binding between OMV^PD−L1 scFv^ and recombinant mouse PD-L1–Fc. (**E**) Nano-flow cytometry analysis of PD-L1–Fc binding to vesicles. OMVs were incubated with fluorescently labeled PD-L1–Fc, washed, and analyzed to quantify fluorescent vesicles. (**F, G**) PD-L1-dependent uptake of DiI-labeled OMV^PD−L1 scFv^ by CHO-mPD-L1 cells with or without anti–PD-L1 blocking antibody. Representative fluorescence images (**F**) and flow cytometry quantification (**G**; mean fluorescence intensity; *n* = 3 independent experiments). Scale bars: 50 μm. (**H**) Representative in vivo whole-body fluorescence images of tumor site in A20 tumor-bearing BALB/c mice acquired 24 h after intravenous administration of DiR-labeled unmodified OMVs, isotype control OMVs displaying an irrelevant HER2 scFv (OMV^Her2−scFv^), or OMV^PD−L1 scFv^. (**I, J**) Ex vivo IVIS imaging of tumors 24 h after intravenous injection of DiR-labeled OMVs, OMV^Her2−scFv^ or OMV^PD−L1 scFv^ (**I**) and quantification of average radiance (**J**; *n* = 3 mice per group). (**K**) Time-course quantification of the in vivo average radiance in the tumor regions at 12-, 24-, and 48-hours post-injection, comparing unmodified OMVs and OMV^PD−L1 scFv^. (**L, M**) Ex vivo IVIS imaging of organs after intravenous injection of DiR-labeled OMVs, OMV^Her2−scFv^ or OMV^PD−L1 scFv^ (**L**) and quantification of average radiance (**M**; *n* = 3 mice per group). Data are mean ± SEM. Statistical significance was calculated using one-way ANOVA with Tukey’s multiple comparisons test (**J**) or Student’s t-test (**G, K**). *P < 0.05, **P < 0.01; ns, not significant

Immunoblotting of bacterial lysates and purified vesicles confirmed expression and enrichment of the ClyA-PD-L1scFv fusion protein in OMV^PD−L1 scFv^ **(**Fig. 1A**)**. Transmission electron microscopy and dynamic light scattering showed that parental OMVs and OMV^PD−L1 scFv^ retained comparable spherical morphology and nanoscale size distributions **(**Fig. 1B, C**)**.

We next verified PD-L1 recognition by OMV^PD−L1 scFv^. Co-immunoprecipitation with recombinant mouse PD-L1-Fc (mPD-L1-Fc) and nano-flow cytometry both confirmed specific binding of OMV^PD−L1 scFv^ to PD-L1 **(**Fig. 1D, E**)**. Consistently, uptake of DiI-labeled OMV^PD−L1 scFv^ by CHO cells expressing mouse PD-L1 (CHO-mPD-L1) was reduced by competitive blockade with an anti-PD-L1 antibody, indicating PD-L1-dependent association and internalization **(**Fig. 1F, G**)**.

To rigorously validate that the enhanced in vivo homing is strictly mediated by specific PD-L1 engagement, we engineered an isotype control displaying an irrelevant scFv (OMV^Her2−scFv^) (Supplementary Fig. S2). In an A20 tumor-bearing BALB/c model, DiR-labeled OMV^PD−L1 scFv^ demonstrated a significantly increased tumor accumulation at 24 h compared to both unmodified OMVs and the OMV^Her2−scFv^ isotype control (Fig. 1H–J). Time-course analysis further revealed a sustained and superior retention of OMV^PD−L1 scFv^ in the tumor site up to 48 h (Fig. 1K, Supplementary Fig. S3). Importantly, ex vivo imaging of major organs confirmed that while all OMV formulations exhibited the expected reticuloendothelial system clearance (primarily in the liver and spleen), the PD-L1-targeted modification did not induce any overt increase in off-target systemic accumulation (Fig. 1L, M).

### OMV^PD−L1 scFv^ enhances DC co-stimulation and reprograms macrophages to a pro-inflammatory phenotype

**Fig. 2:**
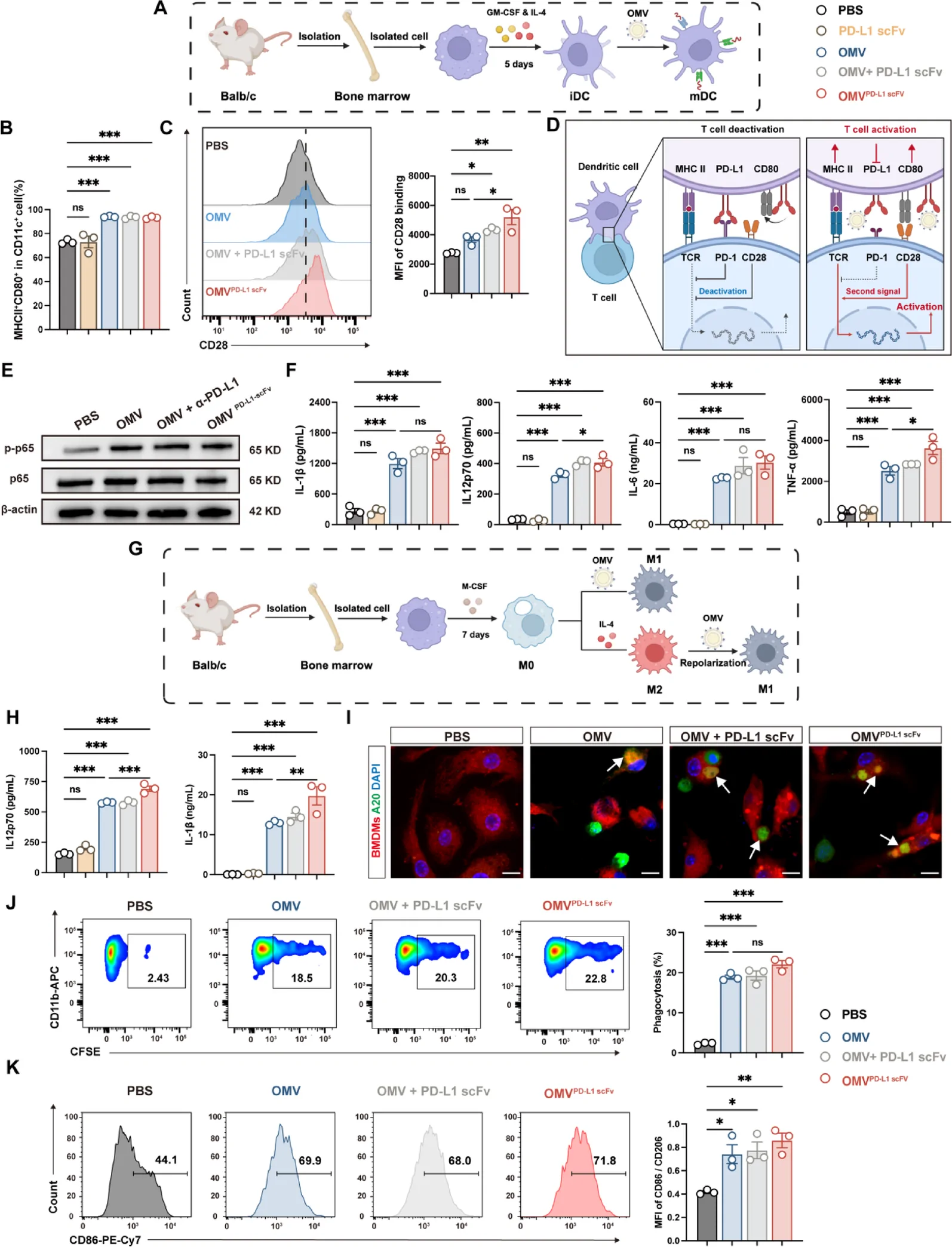
OMV^PD−L1 scFv^ enhances DC co-stimulation and reprograms macrophages to promote tumor phagocytosis.** (A)** Schematic of BMDC differentiation and stimulation with the indicated formulations. **(B)** Flow cytometry quantification of mature BMDCs (MHC-II^+^CD80^+^ among CD11c^+^ cells) after treatment with PBS, soluble PD-L1 scFv, OMVs, OMVs plus scFv, or OMV^PD−L1 scFv^ (*n* = 3 independent experiments). **(C)** Binding of recombinant CD28–Fc to treated BMDCs measured by flow cytometry (anti-human Fc detection); representative histograms (left) and mean fluorescence intensity (right) (*n* = 3). **(D)** Model illustrating how OMV^PD−L1 scFv^ can enhance CD28–CD80 co-stimulation by inducing CD80 and blocking PD-L1 on antigen-presenting cells. **(E)** Immunoblot of NF-κB p65 phosphorylation in BMDCs after treatment with the indicated formulations. **(F)** Cytokines concentration in BMDC culture supernatants (IL-1β, IL-12p70, IL-6, and TNF-α; *n* = 3). **(G)** Schematic of BMDM differentiation and M2 polarization followed by repolarization with OMV formulations. **(H)** Cytokines concentration in BMDM culture supernatants (IL-12p70 and IL-1β; *n* = 3). **(I)** Representative confocal images of BMDM phagocytosis of CFSE-labeled A20 tumor cells (arrows). BMDMs are labeled with CellTrace Red CMTPX and nuclei with DAPI. Scale bars: 10 μm. **(J)** Flow cytometry quantification of phagocytosis (CFSE^+^ events among CD11b^+^ cells) after BMDMs were treated with the indicated formulations (10 µg/mL) and co-cultured with CFSE-labeled A20 cells (*n* = 3). **(K)** Flow cytometry analysis of CD86 on IL-4-polarized M2-like BMDMs after OMV treatment (10 µg/mL) and quantification of the CD86/CD206 mean fluorescence intensity ratio (*n* = 3). Data are mean ± SEM. Statistical significance was calculated using one-way ANOVA with Tukey’s multiple-comparisons test (B, C, F, H, J, K). *P < 0.05, **P < 0.01, ***P < 0.001; ns, not significant

To define how OMV^PD−L1 scFv^ shapes myeloid immunity, we differentiated bone marrow-derived dendritic cells (BMDCs) using GM-CSF and IL-4 (Fig. 2A). Both OMVs and OMV^PD−L1 scFv^ induced BMDC maturation, increasing expression of antigen presentation and co-stimulatory markers, including MHC-II, CD80, and CD86 (Fig. 2B and Supplementary Fig. S4). Because PD-L1 can limit co-stimulation, we assessed the availability of CD80 for CD28 engagement by measuring binding of recombinant CD28-Fc to treated BMDCs and lymph node cells. Compared with parental OMVs, OMV^PD−L1 scFv^ significantly increased CD28-Fc binding (Fig. 2C and Supplementary Fig. S5), consistent with enhanced co-stimulatory capacity (Fig. 2D). At the signaling level, both OMV formulations activated NF-*κ*B (p65 phosphorylation) and induced secretion of inflammatory cytokines, including IL-1β, IL-12p70, IL-6, and TNF-α (Fig. 2E, F).

We then evaluated macrophage responses. Bone marrow-derived macrophages (BMDMs) were generated with M-CSF and polarized with IL-4 to obtain an M2-like state (Fig. 2G). OMV^PD−L1 scFv^ increased IL-12p70 and IL-1β secretion from BMDMs (Fig. 2H) and enhanced phagocytosis of A20 tumor cells in vitro (Fig. 2I, J). Importantly, OMV^PD−L1 scFv^ also repolarized IL-4-polarized M2 BMDMs toward an M1-like phenotype, as evidenced by an increased CD86/CD206 ratio by flow cytometry (Fig. 2K), upregulation of *Nos2* and downregulation of *Arg1* transcripts (Supplementary Fig. S6). Together, these data indicate that OMV^PD−L1 scFv^ can amplify antigen presentation and co-stimulation while shifting macrophages toward a pro-inflammatory, tumor-phagocytic state in vitro.

### Systemic OMV^PD−L1 scFv^ remodels the TME and inhibits tumor growth with dose-dependent splenic EMH

**Fig. 3:**
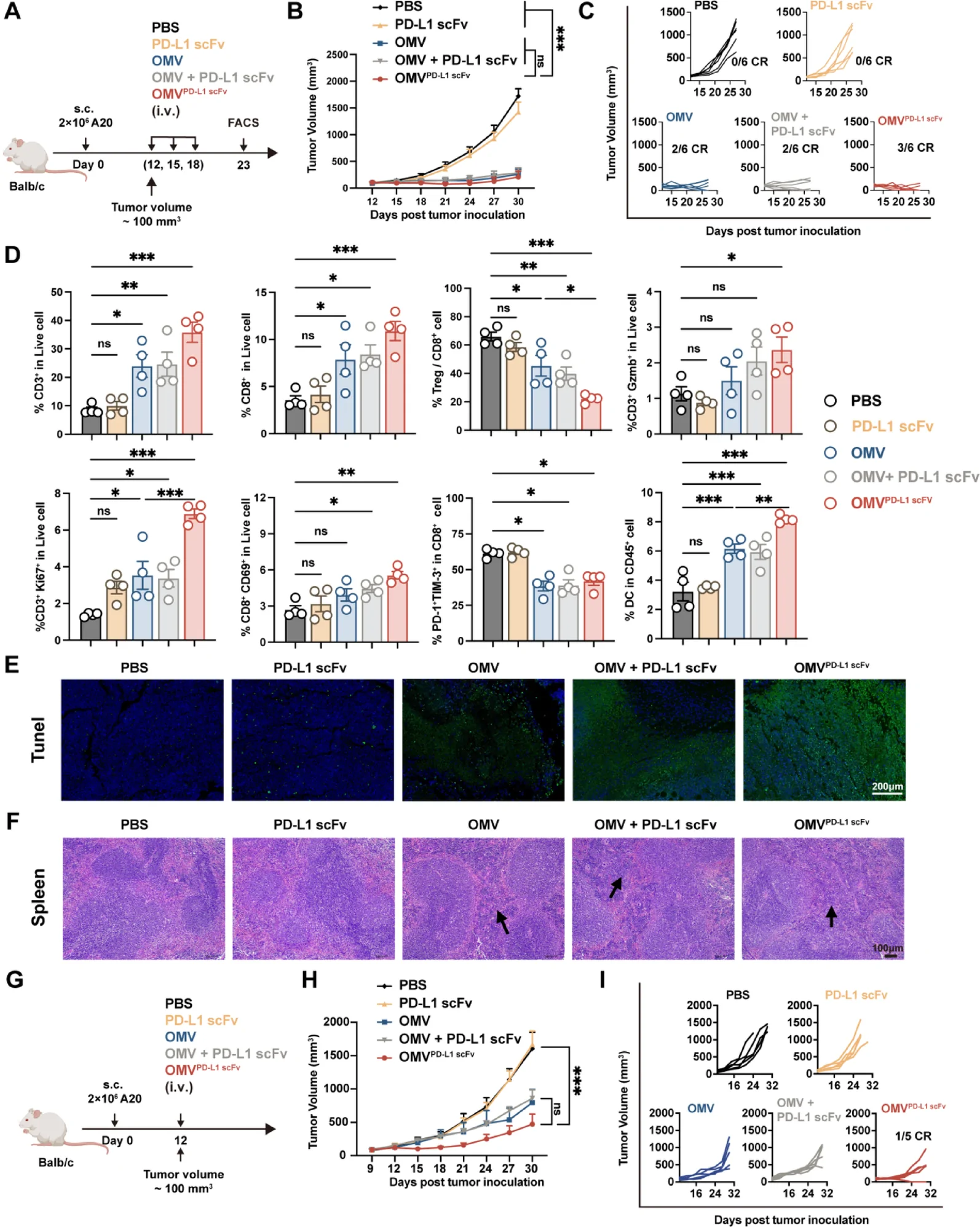
Systemic OMV^PD−L1 scFv^ remodels the tumor microenvironment and inhibits A20 tumor growth. **(A)** Treatment schematic for the syngeneic A20 subcutaneous tumor model in BALB/c mice. When tumors reached ~ 100 mm^3^, mice received intravenous dosing on days 12, 15 and 18 with PBS, soluble PD-L1 scFv, OMVs, OMVs + PD-L1 scFv, or OMV^PD−L1 scFv^ (1 mg/kg; *n* = 6 mice per group). Tumors were harvested for flow cytometry on day 23. **(B)** Mean tumor volume growth curves. **(C)** Individual tumor growth curves. CR, complete remission. **(D)** Flow cytometry quantification of intratumoral immune populations and phenotypes (*n* = 4 mice per group). **(E)** Representative TUNEL staining of tumor sections (green, apoptotic cells) on day 23. Scale bar: 200 μm. **(F)** Representative H&E staining of spleens on day 23; arrows indicate extramedullary hematopoiesis. Scale bar: 100 μm. (G–I) Single-dose regimen in the A20 model **(G)** with mean tumor volume growth curves **(H)** and individual tumor growth curves **(I)** (*n* = 5 mice per group). Data are mean ± SEM. Statistical significance was calculated using one-way ANOVA with Tukey’s multiple-comparisons test (B, D, H). *P < 0.05, **P < 0.01, ***P < 0.001; ns, not significant

We next assessed antitumor activity in syngeneic BALB/c models of A20 lymphoma and CT26 colorectal carcinoma (CRC). In the A20 model, treatment began when tumors reached approximately 100 mm^3^. Mice received three intravenous doses (days 12, 15, and 18) of PBS, soluble PD-L1 scFv, parental OMVs, a mixture of OMVs plus scFv, or OMV^PD−L1 scFv^ **(**Fig. 3A**)**. All OMV-containing regimens delayed tumor growth compared with controls, with OMV^PD−L1 scFv^ achieving the highest complete remission (CR) rate (3/6) relative to OMVs (2/6) or OMVs plus PD-L1 scFv (2/6) **(**Fig. 3B, C**)**.

To link efficacy with immune remodeling, we profiled tumor-infiltrating leukocytes by flow cytometry (Supplementary Fig. S7, 8). Overall, OMV-based treatments significantly increased the intratumoral infiltration of CD3^+^ and CD8^+^ T cells and uniformly reduced the frequency of terminally exhausted PD-1^+^ TIM-3^+^ CD8^+^ T cells compared to the PBS control. Importantly, statistical comparisons revealed that the PD-L1 scFv modification drove specific, significant enhancements over the unmodified OMV chassis. Compared directly to unmodified OMVs, OMV^PD−L1 scFv^ significantly further increased T cell proliferation (Ki67^+^ in CD3^+^ cells) and dendritic cell infiltration, while significantly lowering the immunosuppressive Treg-to-CD8^+^ ratio. Furthermore, while unmodified OMVs failed to induce a statistically significant increase in T cell cytotoxicity (Granzyme B^+^ in CD3^+^ cells) and activation (CD8^+^CD69^+^ T cells) relative to PBS, treatment with OMV^PD−L1 scFv^ successfully elevated both of these functional markers to levels significantly higher than those of the PBS control. (Fig. 3D). OMV^PD−L1 scFv^ produced extensive tumor apoptosis by TUNEL staining (Fig. 3E) and presented significant nuclear condensation and fragmentation on H&E sections (Supplementary Fig. S9A). No abnormalities were detected in the other organs (Supplementary Fig. S9B).

In the more aggressive CT26 CRC model, OMV^PD−L1 scFv^ also improved tumor control and yielded a CR rate of 1/6, whereas parental OMVs produced no CR (Supplementary Fig. S10A-C). However, spleens collected seven days after the last dose showed extramedullary hematopoiesis (EMH) in both models after the three-dose regimen (Fig. 3F and Supplementary Fig. S10D), prompting us to test a single-dose schedule. A single intravenous dose of OMV^PD−L1 scFv^ significantly delayed A20 tumor growth but achieved fewer complete remissions and permitted late tumor regrowth (Fig. 3G-I). Importantly, systemic administration of a single dose of OMV^PD−L1 scFv^ was well tolerated. The transient, early-stage cytokine elevation reflected effective acute immune priming without triggering deleterious hyperinflammation. Consistent with this controlled response, mice exhibited only a mild, reversible weight reduction at day 1. Furthermore, EMH and splenomegaly were mild and transient in the single-dose setting. They completely resolved by day 7, accompanied by full recovery of body weight, blood counts, and serum biochemistry to levels matching those of healthy controls (Supplementary Figs. S11 and S12). These results indicate a dose-efficacy trade-off and support the use of single-dose OMV^PD−L1 scFv^ as a tolerable priming regimen for combination therapy.

### OMV^PD−L1 scFv^ enhances DC-assisted CAR-T activation, tumor killing and trafficking

Given that OMV^PD−L1 scFv^ activates antigen-presenting cells and alleviates immunosuppression, we asked whether OMV priming could augment CAR-T responses. We used an anti-mouse CD19 CAR-T (αmCD19-CAR-T) system and incorporated dendritic cells to mimic antigen presentation in vitro.

**Fig. 4:**
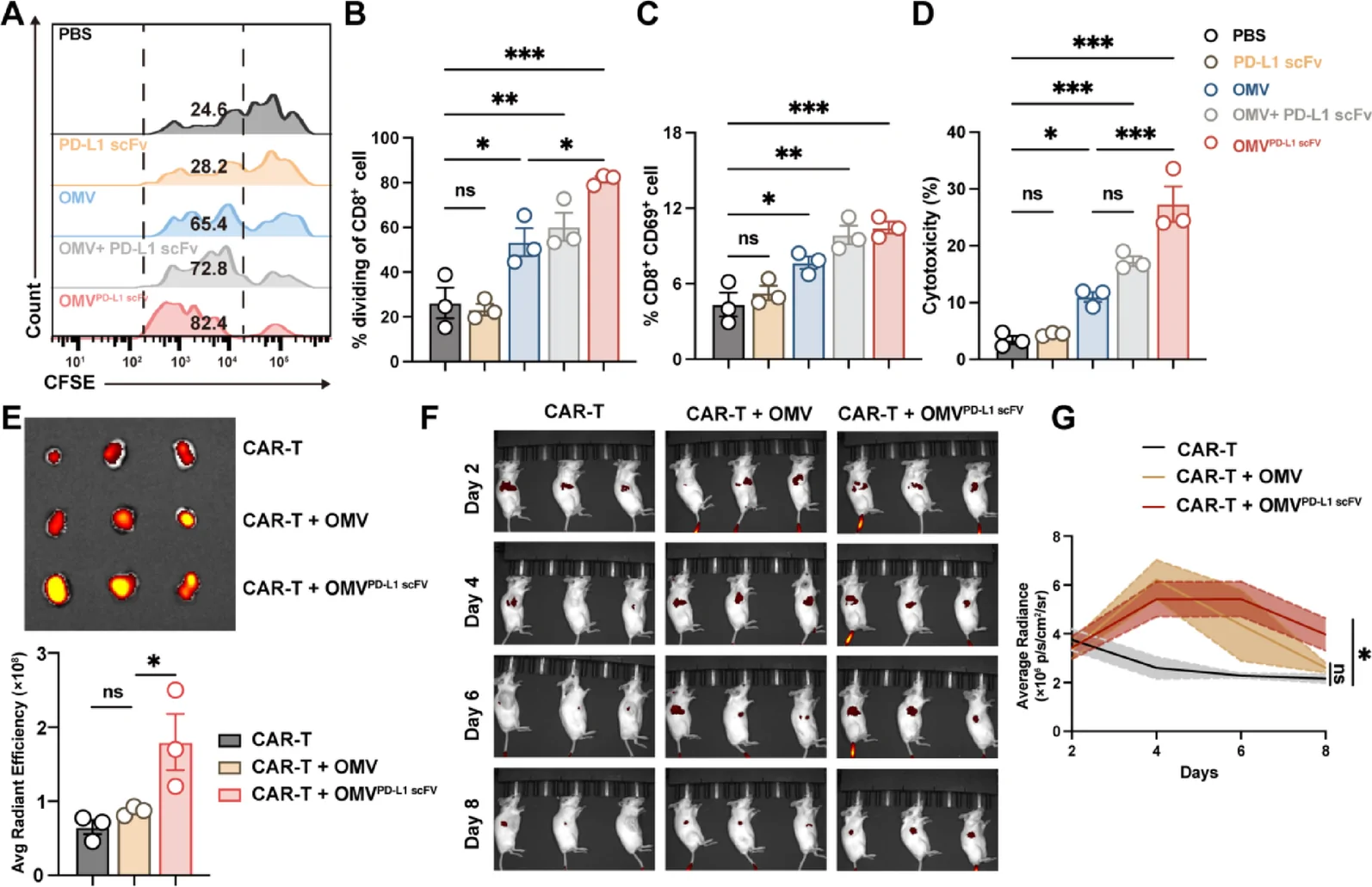
OMV^PD−L1 scFv^ enhances CAR-T activation, cytotoxicity and tumor trafficking**.** BMDC-containing tri-cell co-culture with CD19^+^ luciferase-expressing tumor cells (CT26-mCD19-Luc), CFSE-labeled αmCD19-CAR-T cells and BMDCs. **(A)** Representative CFSE histograms. **(B)** Quantification of dividing CD8^+^ CAR-T cells. **(C)** CD69 expression on CD8^+^ CAR-T cells. **(D)** Tumor cell lysis by luciferase readout (*n* = 3 independent experiments). (**E**) IVIS images(upper) and statistical quantification (bottom) of DIR signals of tumor in ex vivo tumor recruitment assay. Tumors were isolated from CT26-mCD19-bearing mice pretreated with PBS, OMVs or OMV^PD−L1 scFv^ and then co-cultured with DiR-labeled CAR-T cells and imaged (*n* = 3). **(F)** In vivo IVIS imaging after intravenous infusion of DiR-labeled CAR-T cells into CT26-mCD19 tumor-bearing mice preconditioned with PBS, OMVs or OMV^PD−L1 scFv^. (**G**) Quantification of tumor-associated DiR signal over time (*n* = 3 mice per group). Data are mean ± SEM. Statistical significance was calculated using one-way ANOVA with Tukey’s multiple-comparisons test (**B–E, G**). *P < 0.05, **P < 0.01, ***P < 0.001; ns, not significant

In a tri-cell co-culture containing CT26-mCD19-Luc tumor cells, BMDCs, and CFSE-labeled αmCD19-CAR-T cells, OMV^PD−L1 scFv^ significantly increased CAR-T proliferation (CFSE dilution) and activation (CD69) compared with parental OMVs or soluble scFv, and enhanced tumor cell lysis assessed by luciferase activity **(**Fig. 4A-D**)**. To probe trafficking, tumors from CT26-mCD19-bearing mice pretreated with OMV formulations were co-cultured with DiR-labeled αmCD19-CAR-T cells. OMV^PD−L1 scFv^ pretreatment increased CAR-T accumulation within the tumor tissue ex vivo **(**Fig. 4E**)**.

Consistently, in vivo IVIS imaging following intravenous infusion of DiR-labeled CAR-T cells showed greater tumor localization and persistence in mice preconditioned with OMV^PD−L1 scFv^ compared with CAR-T alone **(**Fig. 4F, G**)**. Notably, while the unmodified OMVs showed a visual trend of early recruitment, statistical analysis revealed they failed to induce a significant overall improvement in in vivo CAR-T accumulation relative to CAR-T alone. Together, these data support the use of OMV^PD−L1 scFv^ as an effective immunological primer that enhances CAR-T activation and improves tumor trafficking.

### OMV^PD−L1 scFv^ enables low-dose CAR-T therapy in advanced CT26-mCD19 CRC

**Fig. 5:**
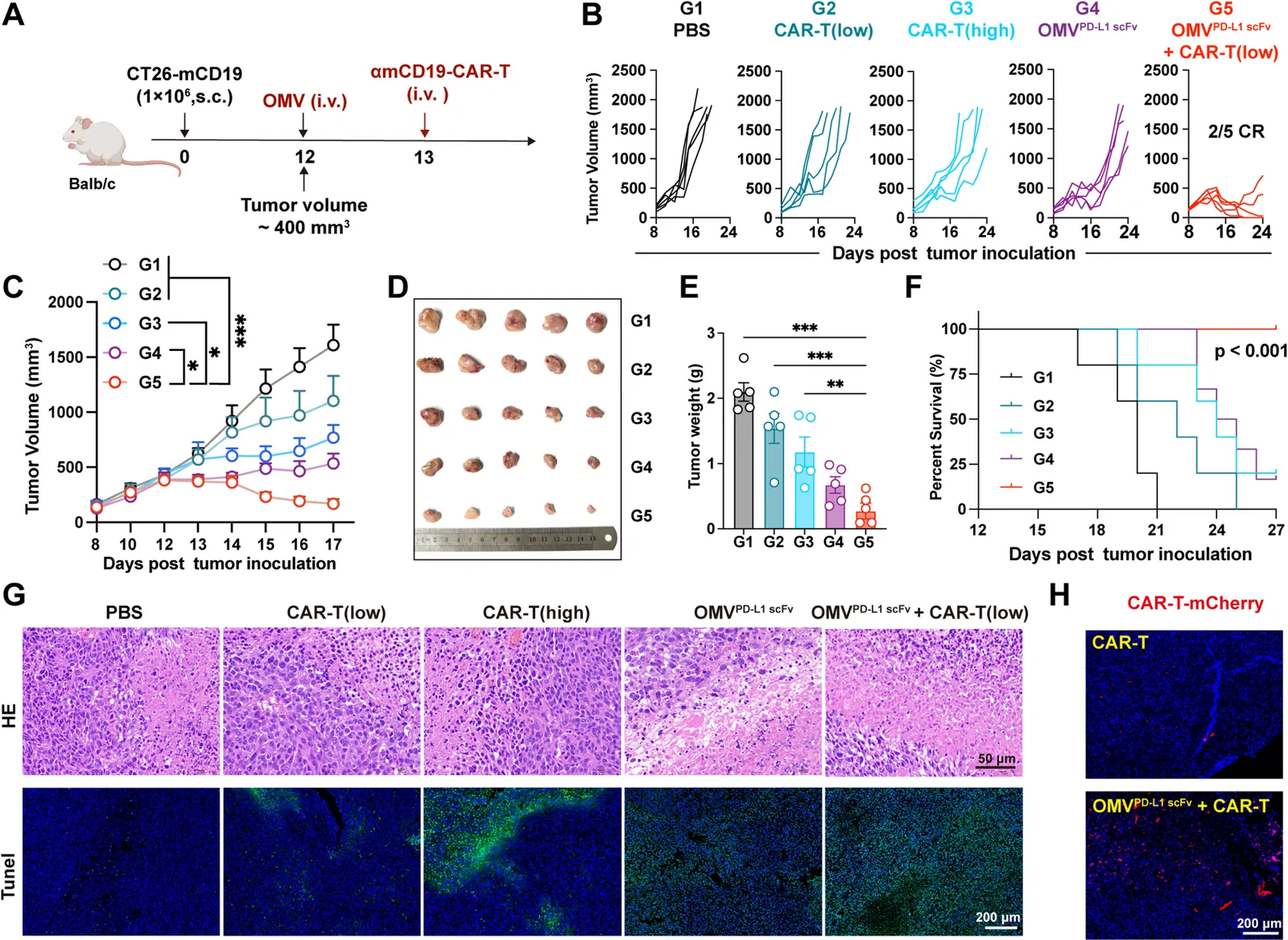
OMV^PD−L1 scFv^ enables low-dose CAR-T control of advanced CT26-mCD19 tumors. (**A**) Treatment schematic in the advanced-stage CT26-mCD19 model (tumor volume ~ 400 mm^3^ at initiation). Mice received OMV^PD−L1 scFv^ (1 mg/kg, i.v.) followed by αmCD19-CAR-T infusion. (**B**) Individual tumor growth curves. (**C**) Mean tumor volume growth curves (*n* = 5 mice per group). (**D**) Tumor images on the 18th day. (**E**) Tumor weights. (**F**) Kaplan–Meier survival analysis (*n* = 5). (**G**) Representative H&E (top) and TUNEL (bottom) staining of tumors at endpoint. Scale bars: 50 μm (**H&E**) and 200 μm (TUNEL). (**H**) Representative tumor-edge infiltration of mCherry-labeled CAR-T cells three days after infusion with or without OMV^PD−L1 scFv^ preconditioning. Scale bar: 200 μm. Data are mean ± SEM. Statistical significance was calculated using one-way ANOVA with Tukey’s multiple-comparisons test (C, E) and the log-rank (Mantel–Cox) test (F). *P < 0.05, **P < 0.01, ***P < 0.001

To evaluate therapeutic synergy in established solid tumors, we used the CT26-mCD19 model and initiated treatment when tumors reached approximately 400 mm^3^. Mice received OMV^PD−L1 scFv^ (1 mg/kg, i.v.) followed 24 h later by either low-dose (1 × 10^6^) or high-dose (5 × 10^6^) αmCD19-CAR-T cells (Fig. 5A, Supplementary Fig. S13A). All active regimens delayed tumor progression relative to PBS; however, OMV^PD−L1 scFv^ combined with low-dose CAR-T produced the most durable control, achieving complete tumor regression in 2/5 mice and significantly prolonging survival (Fig. 5B-F). Gross tumor images and endpoint tumor weights corroborated superior tumor control in the combination group (Fig. 5D, E). Histological analysis revealed widespread tumor destruction and apoptosis in the combination group, as assessed by H&E and TUNEL staining (Fig. 5G). Serum biochemistry at endpoint did not indicate additional systemic toxicity and was consistent with mitigation of advanced tumor-associated abnormalities (Supplementary Fig. S14).

Finally, using CAR-T cells co-expressing mCherry, we observed increased tumor-edge infiltration 3 days after infusion in mice preconditioned with OMV^PD−L1 scFv^ **(**Fig. 5H**)**, supporting enhanced trafficking as a key contributor to efficacy.

### OMV^PD−L1 scFv^ increases intratumoral CAR-T accumulation and reshapes myeloid immunity in advanced hepatocellular carcinoma

**Fig. 6:**
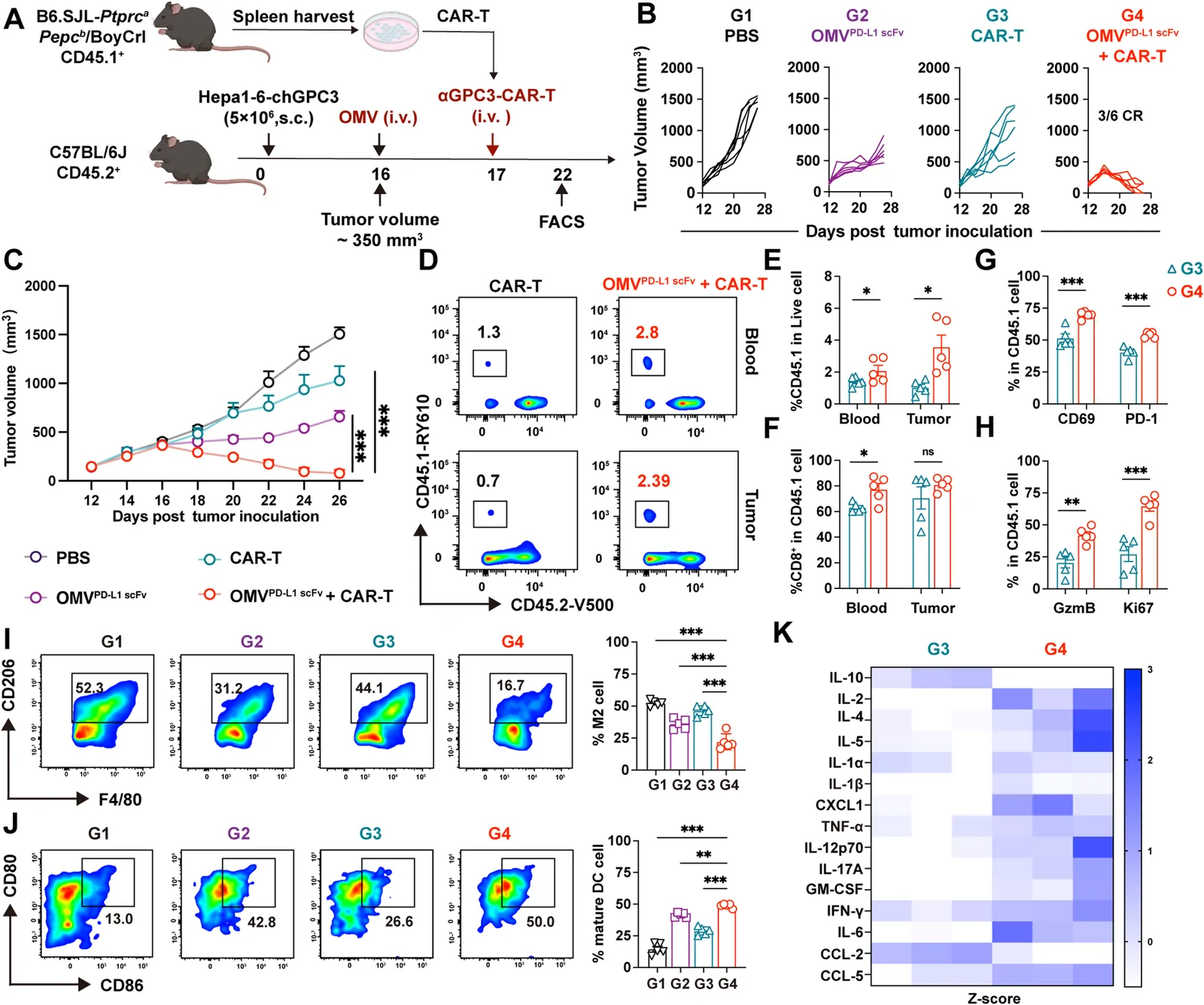
OMV^PD−L1 scFv^ enhances CAR-T accumulation and reshapes myeloid immunity in advanced hepatocellular carcinoma. (**A**) Treatment schematic for the Hepa1-6-chGPC3 syngeneic model in C57BL/6 mice (CD45.2^+^ hosts) using congenic CD45.1^+^ αGPC3-CAR-T cells. (**B**) Individual tumor growth curves. (**C**) Mean tumor volume growth curves (*n* = 6 mice per group). (**D**) Representative flow cytometry plots of CD45.1^+^ and CD45.2^+^ staining in the peripheral blood and tumor (gate on live cells). (**E, F**) Quantification of CD45.1^+^ CAR-T cells among live cells in blood and tumors (**E**) and of CD8^+^ cells within the CD45.1^+^ compartment (**F**) (*n* = 5). **(G)** The percentage of CD8^+^ CD69^+^ and CD8^+^ PD-1^+^ in CD45.1^+^ (*n* = 5). **(H)** The percentage of CD8^+^ Ki67^+^ and CD8^+^ GzmB^+^ (*n* = 5). (**I, J**) Representative plots (left) and quantitative analysis (right) of M2-macrophages (**I**) and mature DCs (**J**) in the tumors. (**K**) Serum cytokine and chemokine heat map at day 19 (*n* = 3). Data are mean ± SEM. Statistical significance was calculated using t-test (E-H) and one-way ANOVA with Tukey’s multiple-comparisons test (**C, I **and** J**). *P < 0.05, **P < 0.01, ***P < 0.001; ns, not significant

To test generalizability across tumor types and antigens, we established a syngeneic Hepa1-6-chGPC3 hepatocellular carcinoma model in C57BL/6 mice. Congenic CD45.1^+^ T cells were transduced with a GPC3-targeting CAR (αGPC3-CAR-T) and infused into CD45.2^+^ tumor-bearing hosts to enable tracking (Fig. 6A, Supplementary Fig. S13B). When tumors reached approximately 350 mm^3^, OMV^PD−L1 scFv^ preconditioning followed by αGPC3-CAR-T therapy achieved complete tumor regression in 3/6 mice, whereas progressive disease occurred in all monotherapy groups (Fig. 6B, C). Upon re-challenge with Hepa1-6-chGPC3 cells on day 60, cured mice exhibited complete tumor rejection. This demonstrates that potent, long-term immunological memory against tumor recurrence was established (Supplementary Fig. S15).

Flow cytometry revealed that OMV^PD−L1 scFv^-containing regimens increased the frequency of CD45.1^+^ CAR-T cells within tumors and improved persistence of circulating CAR-T cells (Fig. 6D-F). Furthermore, intratumoral CAR-T cells in the combination group exhibited a more activated, cytotoxic, and proliferative phenotype, characterized by elevated expression of PD-1, CD69, granzyme B, and Ki67 (Fig. 6G, H, Supplementary Fig. S16).

In parallel, OMV^PD−L1 scFv^ reduced M2-like macrophages (F4/80^+^CD206^+^) and increased mature antigen-presenting cells (CD80^+^CD86^+^) (Fig. 6I, J). To determine whether this local APC activation successfully contributed to T-cell priming in lymph nodes, we profiled tumor-draining and non-draining lymph nodes, with a specific focus on comparing the CAR-T monotherapy and the OMV^PD−L1 scFv^ + CAR-T combination group (Supplementary Fig. S17). Flow cytometry revealed that OMV^PD−L1 scFv^ significantly promoted the accumulation of mature, migratory dendritic cells (CD103^+^ CCR7^+^) within the tdLN (Supplementary Fig. S18A–D). Consequently, this orchestrated a robust and spatially restricted T-cell priming cascade; we observed a profound expansion of IFN-γ-producing tumor-specific T cells and effector memory (Tem) subsets specifically in the tdLN, whereas the contralateral ndLN remained immunologically quiescent (Supplementary Fig. S18E–I). Serum cytokine and chemokine profiling further indicated induction of immune recruitment and effector programs in mice receiving OMV^PD−L1 scFv^ + CAR-T (Fig. 6K).

Collectively, these findings indicate that OMV^PD−L1 scFv^ can remodel the tumor microenvironment to support CAR-T accumulation and function in advanced solid tumors.

### scRNA-seq reveals early immune remodeling and rewired myeloid–lymphoid crosstalk following OMV^PD−L1 scFv^ priming

**Fig. 7:**
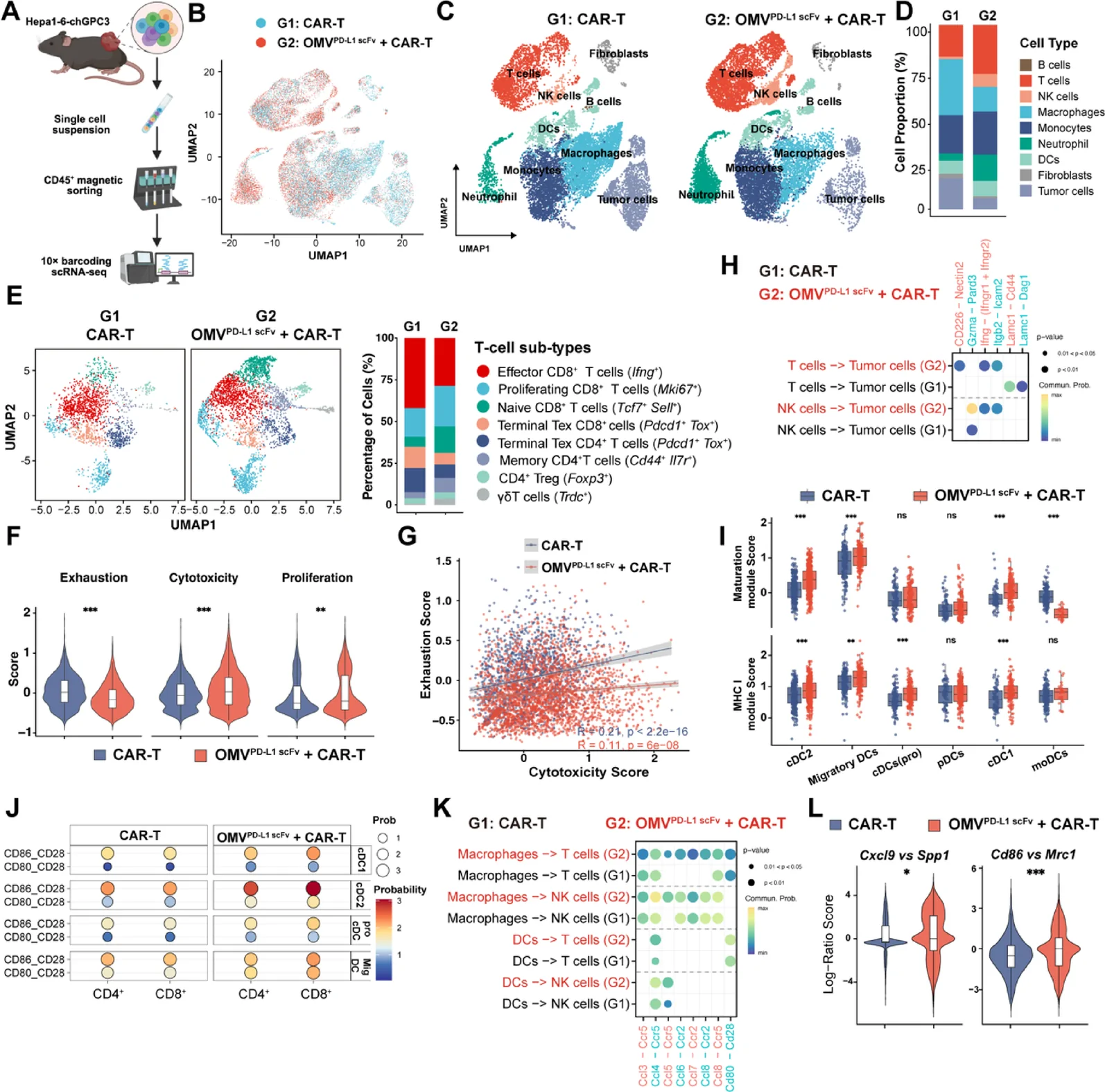
scRNA-seq defines immune remodeling and myeloid–lymphoid crosstalk induced by OMV^PD−L1 scFv^ priming. (**A**) Experimental workflow for single-cell RNA sequencing of dissociated Hepa1-6-chGPC3 tumors collected 5 days after CAR-T infusion (CAR-T versus OMV^PD−L1 scFv^ + CAR-T). (**B**) Merged UMAP of cell clusters from intratumoral CD45^+^ immune cells and tumor cells between two groups. (**C**) Split UMAP including 9 cell clusters (CAR-T: 11,720 cells; OMV^PD−L1 scFv^ + CAR-T: 13,012 cells). (**D**) The proportions of individual cell populations identified in tumors. (**E**) Re-clustered UMAP of CD3^+^ T cells showing eight transcriptionally distinct T cell states and their relative abundances across groups. (**F**) Module scores in tumor-infiltrating T cells for exhaustion, cytotoxicity and proliferation. Exhaustion genes: *Pdcd1*, *Havcr2*, *Lag3*, *Tigit*, *Ctla4*, *Tox*, and *Cd101*; cytotoxicity genes: *Gzmb*, *Gzma*, *Prf1*, *Nkg7*, *Gnly*, and *Ifng*; proliferation genes: *Mki67*, *Top2a*, *Pcna*, *Stmn1*, and *Mcm6*. (**G**) Correlation between cytotoxicity and exhaustion scores. Solid lines indicate linear regression fits, and shaded areas represent the 95% confidence intervals. Pearson correlation coefficients (*R*) and *p*-values are displayed for each group. **(H)** Analysis of specific cytotoxicity and adhesion-related signaling pairs from effector cells (T/NK cells) to tumor cells. (**I**) Dendritic cell module scores for maturation (genes: *Ccr7*, *Cd83*, *Lamp3*, *Cd80*, *Cd86*, *Cd40*, and *Fscn1*) and MHC I antigen presentation (genes: *H2-K1*, *H2-D1*, *B2m*, *Tap1*, and *Tap2*). (**J**) CellChat-inferred co-stimulatory ligand–receptor communication probabilities from dendritic cell subsets to CD4^+^ and CD8^+^ T cells *via Cd86–Cd28* and *Cd80–Cd28*. (**K**) CellChat-inferred chemokine communication probabilities from myeloid cells (DCs and macrophages) to T/NK cells highlighting recruitment *via* the *Ccl3/4/5–Ccr5* axis. (**L**) Macrophage polarization log-ratio scores comparing canonical M1 versus M2 programs (*Cxcl9*/*Spp1* and *Cd86*/*Mrc1*). Statistical significance was determined using a two-sided Wilcoxon rank-sum test (F, I, L). *P < 0.05, **P < 0.01, ***P < 0.001; ns, not significant

To complement the flow-cytometry-defined immune remodeling in the advanced Hepa1-6-chGPC3 model **(**Fig. 6**)**, we performed single-cell RNA sequencing on dissociated Hepa1-6-chGPC3 tumors collected 5 days after CAR-T infusion from mice treated with CAR-T alone or OMV^PD−L1 scFv^ preconditioning followed by CAR-T **(**Fig. 7A**)**. After quality control and doublet removal, we obtained 11,720 cells in the CAR-T group and 13,012 cells in the OMV^PD−L1 scFv^ + CAR-T group.

Unsupervised clustering identified nine major populations spanning malignant cells and immune subsets (Fig. 7B, C; Supplementary Fig. S19A, B). OMV^PD−L1 scFv^ priming led to a higher fraction of lymphoid cells, primarily T cells and NK cells, and a lower abundance of malignant cells compared to CAR-T treatment alone. (Fig. 7C, D). Furthermore, differential expression followed by Gene Ontology (GO) and Kyoto Encyclopedia of Genes and Genomes (KEGG) enrichment analyses revealed an upregulation of transcriptomic signatures associated with inflammatory and cytotoxic pathways in the combination group (Supplementary Fig. S19C, D). CellChat predicted a global increase in inferred cell-cell communications in the OMV^PD−L1 scFv^ + CAR-T combination group compared with the CAR-T group. Notably, macrophages and DCs showed significantly enriched transcript profiles for outgoing signaling pathways targeting NK cells and T cells (Supplementary Fig. S19E).

Taken together, the combination therapy fundamentally remodels the immune landscape, driving a marked shift from myeloid dominance to robust lymphocyte infiltration.

### OMV^PD−L1 scFv^ expands proliferative, stem-like cytotoxic T cell states while constraining terminal exhaustion

To determine whether transcriptional signatures of functional enhancement accompanied this increased infiltration, we further examined the intrinsic transcriptional states of the lymphocytes. Differential expression analysis of tumor-infiltrating immune cells revealed that combination therapy is associated with a transcriptional shift in T and NK cells toward a hyper-functional, non-exhausted profile, characterized by the upregulation of activation (*Cd69*, *Klrk1*), cytotoxicity (*Gzma*, *Gzmb*, *Prf1*), and stem-like memory genes (*Tcf7*, *Eomes*, *Bcl2*), alongside the marked downregulation of inhibitory receptors such as *Lag3*, *Tigit*, and *Havcr2*. And significant upregulation of *Xcl1* suggests a potential transcriptomic mechanism for the recruitment of cDC1s into the tumor niche (Supplementary Fig. 20A). Consistent with tumor regression, proliferation markers (*Mki67*, *Top2a*) were significantly enriched in lymphocytes but diminished in malignant cells. (Supplementary Fig. 20B).

Re-clustering of CD3^+^ T cells resolved eight transcriptionally distinct states (Fig. 7E; Supplementary Fig. S20C). Notably, the combination therapy significantly depleted terminally exhausted and immunosuppressive subsets while expanding the pools of proliferating and Naive /stem-like CD8^+^ T cells (Fig. 7E). Module scoring corroborated these shifts at the population level: T cells in the combination group exhibited lower exhaustion scores together with higher cytotoxicity and proliferation scores compared with CAR-T alone (Fig. 7F). Notably, the relationship between cytotoxicity and exhaustion was attenuated in OMV^PD−L1 scFv^ + CAR-T tumors, with cytotoxic programs maintained without a proportional increase in exhaustion signatures (Fig. 7G). Meanwhile, inferential cell-cell communication analysis of the interaction between NK/T cells and tumor cells revealed that the combination group exhibited enhanced predicted signaling of cytotoxicity (*Ifng*-(*Ifngr1 + Ifngr2*) & *Gzma-Pard3*) and transcriptomic evidence of restored activating synapses (*Cd226-Nectin2*,* Itgb2-Icam1*) that replaced inhibitory signals. These transcriptomic alterations are consistent with enhanced tumor lysis and coincided with a compromised tumor niche structure (*Lamc1-Cd44/Dag1*) (Fig. 7H).

Together, these data indicate that OMV^PD−L1 scFv^ priming supports an expanded pool of proliferative, stem-like cytotoxic T cells while limiting progression toward terminal exhaustion.

### Myeloid licensing and chemokine-driven recruitment underpin enhanced co-stimulation in the tumor niche

Given the prominent lymphoid accumulation, we asked how the transcriptomes of myeloid compartments were altered to support recruitment and sustained activation potentially. We performed unsupervised sub-clustering of the DC compartment. Quantitative analysis demonstrated that the combination therapy significantly remodeled the DC landscape, leading to a marked expansion of conventional dendritic cells (cDCs) compared to the CAR-T control (Supplementary Fig. 21A-C). Dendritic cells displayed higher maturation and MHC I antigen-presentation transcriptional module scores in the combination group (Fig. 7I), consistent with a more competent antigen-presenting niche. CellChat analysis further predicted strengthened co-stimulatory communication from DC subsets to CD4^+^ and CD8^+^ T cells through CD80–CD28 and CD86–CD28 ligand–receptor pairs (Fig. 7J, Supplementary Fig. S21D), supporting a transcriptional basis for enhanced “signal 2” delivery within the tumor microenvironment. In parallel, transcripts encoding myeloid-to-lymphoid recruitment cues were selectively enriched. In particular, CellChat highlighted increased inferred chemokine signaling from macrophages and DCs to T/NK cells *via* the *Ccl3/4/5–Ccr5* axis (Fig. 7K), suggesting a transcriptomic basis for effector recruitment into the tumor core.

Finally, when analyzing the relative dominance of these states within tumor-associated macrophages (TAMs), we observed that macrophage transcriptional signatures were shifted toward a more inflammatory, antitumor state. (Supplementary Fig. S21E-G). Ratio-based scoring revealed an increased M1-like transcriptional dominance relative to M2-like programs, including higher *Cxcl9*/*Spp1* and *Cd86*/*Mrc1* log-ratios (Fig. 7L). This transcriptional shift was accompanied by metabolic pathway enrichment, with macrophages exhibiting increased glycolysis and altered oxidative phosphorylation signatures (OXPHOS) gene signatures (Supplementary Fig. S21H, I). Collectively, these single-cell transcriptomic analyses support a model in which OMV^PD−L1 scFv^ priming is associated with myeloid licensing signatures that predict enhanced effector lymphocytes and enhanced co-stimulatory signaling, thereby sustaining robust CAR-T-associated cytotoxic transcriptional programs while limiting terminal exhaustion signatures.

## Discussion

In this study, we developed a PD-L1-targeted bacterial outer membrane vesicle platform (OMV^PD−L1 scFv^) as a tumor-directed priming strategy for CAR-T therapy in solid tumors. Across syngeneic tumor models, OMV^PD−L1 scFv^ improved tumor localization, licensed antigen-presenting compartments with increased co-stimulation, and reshaped the myeloid landscape toward an immunostimulatory state (Figs. 2 and 3). These changes enhanced CAR-T activation, trafficking, and intratumoral retention (Fig. 4), translating into robust control of established tumors at reduced CAR-T doses (Fig. 5) and generalizability across antigen contexts (Fig. 6). Single-cell transcriptomics further revealed coordinated lymphoid–myeloid rewiring, including stem-like cytotoxic T-cell programs and chemokine-driven recruitment circuits, together with reduced physical-barrier signatures (Fig. 7).

A key conceptual advance of this work is that a “non-cell-intrinsic” priming layer can lower the effective threshold for adoptive cell therapy. CAR-T efficacy often scales with effector cell fitness and tumor exposure. Still, dose escalation can be constrained by manufacturing burden and clinically relevant toxicities associated with CAR-T therapies [37]. Rather than further intensifying CAR designs, OMV^PD−L1 scFv^ conditions the tumor niche to support efficient effector recruitment and durable function, enabling therapeutic activity even at low CAR-T input (Fig. 5). This complements existing T cell–engineering approaches, such as cytokine/chemokine-armored CAR-T cells or chemokine receptor rewiring, that aim to enhance persistence and trafficking [25, 26].

Delineating the contributions of the OMV and the OMV^PD−L1 scFv^ is critical for this platform’s rational design. While unmodified OMVs provide intrinsic adjuvanticity for baseline myeloid engagement, synergistic CAR-T efficacy strictly requires the engineered OMV^PD−L1 scFv^. Rather than merely enhancing tumor accumulation, OMV^PD−L1 scFv^ remodels the immune synapse to overcome local suppression. Biochemically, OMV^PD−L1 scFv^ shields PD-L1, restoring critical “Signal 2” by significantly enhancing CD28 co-stimulatory binding on antigen-presenting cells (Fig. 2C). This directly drives robust in vitro CAR-T proliferation and tumor killing (Fig. 4). Importantly, this functional advantage translates in vivo into a profound and sustained intratumoral accumulation of CAR-T cells (Fig. 4E-G). This persistence may be driven by a dual-arm mechanism: the reinforced DC co-stimulation actively amplifies CAR-T expansion, while the concurrent scFv-mediated checkpoint blockade protects these recruited lymphocytes from rapid exhaustion. Thus, while the OMV chassis initiates baseline immune recruitment, the PD-L1 scFv on the OMV surface may be a prerequisite for sustaining CAR-T proliferation and ensuring functional persistence.

Mechanistically, our data support a model of a self-reinforcing myeloid–lymphoid circuit linking innate activation to sustained effector competence. Transcriptomic profiling and flow cytometry converged on an expansion of functional cytotoxic lymphocytes alongside signatures of increased antigen-presenting and co-stimulatory capacity (Figs. 2, 4, 6 and 7). Notably, lymphoid programs with increased *Xcl1*/*Ccl5* signatures and transcriptional states associated with activated DC states are consistent with prior work showing that NK/T cell-derived XCL1/CCL5 can recruit XCR1^+^ cDC1 and promote intratumoral immune control [38, 39]. In parallel, enrichment of *Ccr5*-associated chemokines within myeloid subsets (Fig. 7K) supports a model in which myeloid compartments amplify lymphocyte recruitment, echoing recent evidence that CCR5-dependent cues can shape monocyte/macrophage dynamics and immunotherapy responses [40]. Importantly, reinforced CD28 co-stimulation provides a plausible molecular bridge between myeloid licensing and the maintenance of stem-like, transcriptionally less exhausted effector states (Fig. 7E–G and J), consistent with the established requirement for CD28 signaling during effective checkpoint-driven reinvigoration [21–24, 41].

Another notable feature of OMV priming is its potential to address the physical constraints of solid tumors. Beyond immunosuppressive signaling, dense stromal architecture can impede immune-cell access and limit CAR-T efficacy, and strategies that alleviate extracellular matrix (ECM) constraints can potentiate adoptive transfer [16]. In our scRNA-seq analyses, OMV^PD−L1 scFv^ preconditioning was associated with a downregulation of transcriptomic signatures linked to tissue remodeling and physical barriers (Fig. 7H and K), coinciding with increased lymphocyte infiltration and retention observed in vivo (Figs. 4, 5 and 6). Given the nanoscale size and bioengineerable surface of OMV-derived platforms, it is plausible that optimized OMV formulations could further improve intratumoral distribution and penetration, as supported by recent reports of OMV-based nanorobotic systems capable of enhanced tissue penetration and tumor delivery [42]. The modification of OMV surface antibodies is highly flexible and largely dictated by expression efficiency in *E. coli*, where smaller antibody formats generally yield better results. Capitalizing on this plasticity, OMVs can be engineered with a smaller PD-L1 nanobody to enhance tumor targeting and checkpoint blockade [43]. Furthermore, loading these OMVs with cytotoxic agents like elesclomol creates a combination therapy that directly kills tumors. This drug-loaded approach contrasts sharply with our “non-cell-intrinsic” strategy, which uses empty, low dose OMVs solely to remodel the tumor microenvironment and prime it for CAR-T cell therapy.

Several limitations should be considered. First, OMVs contain lipopolysaccharide (LPS), which contributes to innate immune activation but can also cause systemic toxicity. Human inflammatory responses to endotoxin differ substantially from those in mice and can be triggered at lower doses, underscoring the translational importance of dose optimization and safety engineering [44]. Second, clinical translation will likely require genetically modified bacterial strains with attenuated lipid A activity or other strategies that reduce reactogenicity while preserving immunostimulatory potency, as demonstrated with detoxified OMV vaccine platforms [45]. A third limitation relates to the direct evaluation of CAR-T-associated toxicities. Although clinical dose de-escalation is motivated in part by the need to reduce systemic toxicities such as CRS, our syngeneic models were not designed to quantify this benefit directly. The “high” CAR-T dose used here represents a therapeutic dose commonly used in preclinical syngeneic studies rather than an intentionally toxic challenge dose, and immunocompetent syngeneic mice do not readily recapitulate dose-limiting CAR-T toxicities. As noted in recent literature, robust modeling of CAR-T side effects often requires specialized models, such as NSG or humanized mice, combined with behavioral scoring, body-temperature monitoring, hemodynamic assessment and cytokine profiling [46]. Future studies using such models will be necessary to determine whether OMV-primed, dose-reduced CAR-T regimens confer a true CRS-sparing or toxicity-sparing benefit. Finally, our models focused on defined tumor antigens and immunocompetent mice; additional work will be needed to evaluate durability across heterogeneous antigen landscapes and to define how OMV priming interfaces with standard therapies and with human tumor ecosystems.

This work also suggests several future directions. First, the coordinated improvement of NK-cell-associated programs observed by scRNA-seq (Fig. 7) raises the possibility that OMV priming could synergize with emerging CAR-NK strategies, which are increasingly being evaluated clinically and preclinically [47, 48]. Second, repeated OMV administration could elicit anti-vesicle or anti-scFv antibody responses that accelerate clearance and limit long-term redosing. An intriguing possibility is to explore autologous or commensal microbiota-derived OMVs, which have been shown to mediate immune modulation in the host, as a route to reduce immunogenicity while retaining programmable delivery features [49]. Together, these findings position tumor-targeted OMV priming as a modular strategy to rewire the solid-tumor TME and to expand the therapeutic window of adoptive cell therapies.

## Supplementary Information

Below is the link to the electronic supplementary material.

Supplementary Material 1.

## Data Availability

No datasets were generated or analysed during the current study.

## Abbreviations

CAR-T: Chimeric antigen receptor T
TME: Tumor microenvironment
OMV: Bacterial outer membrane vesicles
CRC: Colorectal carcinoma
CR: Complete remission
EMH: Extramedullary hematopoiesis
BMDC: Bone marrow-derived dendritic cells
BMDM: Bone marrow-derived macrophages
GO: Gene Ontology
KEGG: Kyoto Encyclopedia of Genes and Genomes
ECM: extracellular matrix
LPS: lipopolysaccharide
MHC: Major Histocompatibility Complex
