# Supplementary material for: PD-L1-targeted OMV priming licenses myeloid co-stimulation and chemokine circuits to potentiate CAR-T therapy in advanced-stage solid tumors
Source: J Nanobiotechnology. 2026 Jun 23;24:834. doi: 10.1186/s12951-026-04722-6 (PMC13543469; doi:10.1186/s12951-026-04722-6)
Supplement: Supplementary file 1 [file 12951_2026_4722_MOESM1_ESM.docx]

**Supplementary Material**

**Materials and Methods**

**Expression and purification of soluble PD-L1 scFv**

The gene sequences encoding PD-L1 scFv were cloned into the pLVX vector to establish CHO stable cell lines. The culture supernatant containing the recombinant protein was filtered through a 0.22 μm membrane (Millipore) to remove cell debris. PD-L1 scFv was purified using HisSep Ni-NTA MagBeads (Yeasen, 20561ES08) and eluted with a buffer containing 50 mM NaH₂PO₄, 300 mM NaCl, and 250 mM imidazole (pH 8.0). Eluted proteins were further purified using a Sephadex Desalting Gravity Column (Sangon Biotech, C500090) and concentrated via ultrafiltration (3 kDa molecular weight cut-off). The purified proteins were aliquoted and stored at -80°C until use. Protein concentrations were determined by using a BCA Protein Assay Kit.

**Western blot analysis for OMV**

Bacteria were lysed in bacterial activity-keeping lysis buffer (C500020, Sangon Biotech). OMVs were lysed in RIPA buffer (P0013B, Beyotime) supplemented with 1 mM PMSF (ST506, Beyotime) on ice. Lysates were clarified by centrifugation (15,000 g, 10 min), and supernatants were resolved by SDS-PAGE and transferred to a 0.22 µm PVDF membrane (ISEQ00010, Millipore). Membranes were blocked in 5% (w/v) skim milk (1172GR100, Biofroxx) in TBST and incubated overnight at 4 °C with anti-6× His HRP-conjugated antibody (1:2,000; ab1187, Abcam).

For co-immunoprecipitation, mouse PD-L1-Fc (CJ89, Novoprotein) was incubated with protein A/G agarose (P2055, Beyotime) for 1 h at room temperature. Beads were washed three times with TBS and incubated with OMV^PD-L1 scFv^ (0.5 mL; 1 mg/mL total protein) overnight at 4 °C. Beads were washed three times with lysis buffer, boiled in SDS loading buffer (95 °C, 5 min), and supernatants were subjected to western blotting. PD-L1 scFv was detected with anti-6 × His HRP antibody (1:2,000; ab1187, Abcam), and PD-L1-Fc was detected with goat anti-mouse IgG-HRP (1:10,000; 31430, Invitrogen).

Immunoreactive proteins were visualized using enhanced chemiluminescence reagents (SQ101, Epizyme), and the luminescent bands were visualized using the ChemiDoc Touch Imaging System (Bio-Rad).

**Cell-based competitive binding and uptake assay**

CHO-mPD-L1 cells were seeded in 12-well plates and pre-incubated with anti-PD-L1 antibody (10 µg/mL) for 1 h at 37 °C where indicated. DiI-labeled OMVs or OMV^PD-L1 scFv^ (10 µg/mL vesicle protein) were then added for 2 h at 37 °C. Cells were washed three times with PBS and analyzed by flow cytometry (LSRFortessa, BD). For fluorescence imaging, nuclei were counterstained with Hoechst 33342 (R37605, Invitrogen) before imaging on a fluorescence microscope (Leica, Germany).

**Cellular uptake assay of DiO-labeled** **OMV^Her2-scFv^**

To evaluate the cellular association and uptake of OMV^Her2-scFv^ by 4T1 and 4T1-hHER2 cells, OMVs were fluorescently labeled with DiO. 4T1 and 4T1-hHER2 cells were seeded into 24-well tissue culture plates and cultured until reaching approximately 60–70% confluence. DiO-labeled OMV^Her2-scFv^ (10 µg/mL vesicle protein) were then added for 4 h at 37 °C. After incubation, cells were gently washed three times with sterile PBS to remove unbound OMVs. Cells were fixed with 4% paraformaldehyde in PBS for 15 min at room temperature. For fluorescence imaging, nuclei were counterstained with DAPI (HY-D2868, MCE) before imaging on a fluorescence microscope.

***In vivo* biodistribution of OMVs**

A20 tumor-bearing female BALB/c mice received DiR-labeled OMVs, OMV^Her2-scFv^ or OMV^PD-L1 scFv^ (10 µg total vesicle protein in PBS, intravenous tail-vein injection). Twenty-four hours after injection, major organs and tumors were harvested and imaged using an IVIS imaging system (PerkinElmer). Images were analyzed using Living Image software (V4.4), and and bioluminescence was quantified as average radiance (p/s/cm^2^/sr).

**BMDC differentiation and activation**

Bone marrow cells were isolated from femurs and tibias of BALB/c mice and cultured in RPMI 1640 supplemented with 10% FBS, GM-CSF (20 ng/mL; CJ46, Novoprotein) and IL-4 (10 ng/mL; CK15, Novoprotein). Half medium was replaced every 2 days. On day 5, immature BMDCs were treated with OMVs, OMVs plus soluble PD-L1 scFv, or OMV^PD-L1 scFv^ (10 µg/mL vesicle protein) for 12 h.

For flow cytometry, BMDCs were stained with FITC-anti-mouse CD11c (clone N418; 11-0114-81, Invitrogen), APC-anti-mouse CD80 (clone 16-10A1; 104714, BioLegend) and PE-Cy7-anti-mouse CD86 (clone GL1; 25-0862-80, Invitrogen).

For NF-κB pathway analysis, BMDC lysates were prepared in RIPA buffer and immunoblotted with anti-NF-κB p65 (1:2,000; 80979-1-RR, Proteintech), anti-phospho-NF-κB p65 (S536) (1:1,000; 3033, Cell Signaling Technology) and anti-β-actin (1:20,000; 660009-1-Ig, Proteintech), followed by HRP-conjugated secondary antibodies (goat anti-mouse IgG: 1:10,000; 31430, Invitrogen; goat anti-rabbit IgG: 1:10,000; 31460, Invitrogen).

**Macrophage phagocytosis assay**

A20 cells were labeled with CFSE (21888, Sigma) according to the manufacturer’s instructions. M0 BMDMs were seeded in 12-well plates and treated with OMVs, OMVs plus PD-L1 scFv, or OMV^PD-L1 scFv^ (10 µg/mL) for 6 h before addition of CFSE-labeled A20 cells (3 × 10^5^). After 12 h of co-culture, wells were washed three times with PBS to remove non-engulfed target cells. BMDMs were stained with PE-eFluor 610-anti-mouse CD11b and analyzed by flow cytometry to quantify CFSE^+^ events within the CD11b^+^ gate.

For confocal imaging, M0 BMDMs were pre-labeled with CellTrace Red CMTPX (40717ES50, Yeasen) and seeded on chamber slides (CS-08-TC, Labselect). After co-culture with CFSE-labeled A20 cells, samples were fixed in 4% paraformaldehyde (BL539A, Biosharp) and nuclei were counterstained with DAPI. Images were acquired by confocal microscopy (LSM880, ZEISS).

**Analysis of M2-BMDMs repolarization**

Bone marrow cells were cultured in DMEM containing 10% FBS, penicillin-streptomycin (100 U/mL), β-mercaptoethanol (0.05 mM; abs9592, Absin) and M-CSF (20 ng/mL; CB34, Novoprotein). Half medium was replaced every 3 days. On day 7, adherent M0 BMDMs were polarized to an M2-like state using IL-4 (40 ng/mL; CK15, Novoprotein) for 48 h. M2-polarized BMDMs were then treated with OMVs, OMVs plus soluble PD-L1 scFv, or OMV^PD-L1 scFv^ (10 µg/mL) for 12 h.

For flow cytometry, Fc receptors were blocked with anti-CD16/CD32 (clone 93; 14-0161-86, Invitrogen) for 20 min at 4 °C and stained with PE-eFluor 610-anti-mouse CD11b (clone M1/70; 61-0112-80, Invitrogen), PE-Cy7-anti-mouse CD86 (clone GL1; 25-0862-80, Invitrogen) and APC-anti-mouse CD206 (clone MMR; 17-2061-82, Invitrogen).

Meanwhile, cells were collected and analyzed M1-associated markers (*iNOS*,) and M2-associated markers (*Arg-1*) by real-time qPCR (RT-qPCR).

**Cytokine measurements by ELISA**

BMDCs or BMDMs were treated with soluble PD-L1 scFv, OMVs, OMVs plus PD-L1 scFv, or OMV^PD-L1 scFv^ (10 µg/mL) for 12 h. Cell-free supernatants were collected and assayed using commercial ELISA kits following the manufacturer’s instructions: TNF-α (BMS607-3, Invitrogen), IL-12p70 (BMS6004, Invitrogen), IL-6 (KMC0061, Invitrogen) and IL-1β (BMS6002-2, Invitrogen).

**Real-time qPCR**

Total RNA was extracted using TRIzol reagent (15596026CN, Invitrogen). cDNA was synthesized using RevertAid Master Mix (M16325, Invitrogen). Quantitative PCR was performed using SsoFast EvaGreen Supermix (1725200, Bio-Rad) on a QuantStudio 3 system (Thermo Fisher). Target gene expression was normalized to *β-actin*, and relative expression was calculated using the Pfaffl method. Primer sequences were as follows: *iNOS* forward, 5’-TGCATGGACCAGTATAAGGCAAGC-3’; *iNOS* reverse, 5’-GCTTCTGGTCGATGTCATGAGCAA-3’; *Arg-1* forward, 5’-AGGAGCTGTCATTAGGGACATC-3’; *Arg-1* reverse, 5’-CTCCAAGCCAAAGTCCTTAGAG-3’; *β-actin* forward, 5’-TTCTTTGCAGCTCCTTCGTT-3’; *β-actin* reverse, 5’- ATGGAGGGGAATACAGCCC-3’.

**Histology and immunofluorescence**

For H&E staining, tumors and major organs (heart, liver, spleen, lung, and kidney) were harvested, fixed in 4% neutral paraformaldehyde and embedded in paraffin. Sections (4 µm) were stained with hematoxylin and eosin and imaged using a Leica DMI 4000B microscope (Wetzlar, Germany).

For visualization of CAR-T-mCherry infiltration, tumors were embedded in OCT compound (4583, Sakura), snap-frozen in liquid nitrogen and cryosectioned (8 µm). Sections were fixed in 4% paraformaldehyde (30 min, room temperature), washed in PBS and counterstained with DAPI. Slides were mounted with antifade mounting medium (P36965, Invitrogen) and images were acquired by a fluorescence microscope (Leica, Germany).

**Multiplex cytokine and chemokine profiling**

Blood samples was collected from Hepa1-6-chGPC3-bearing mice at day 19 and from Balb/c mice at the indicated time points after receiving OMV^PD-L1 scFv^ injection. Serum was obtained by centrifugation (3,000 g, 15 min, 4 °C) and cytokines/chemokines (IL-10, IL-2, IL-4, IL-5, IL-1α, IL-1β, CXCL1, TNF-α, IL-12p70, IL-17A, GM-CSF, IFN-γ, IL-6, CCL2, and CCL5) were quantified using a magnetic bead-based multiplex assay (XMPlex03240771 XMPLEX Mouse macrophage 15-Plex Panel) according to the manufacturer’s instructions with the assistance of Sci-XM Biotechnology Co., Ltd (WuHan, China).

**Hematology and serum biochemistry**

Whole blood was collected into EDTA.2K tubes (G0280, Solarbio) for complete blood counts. Serum was prepared from clotted blood (3,000 g, 15 min) for chemistry testing. ALP, ALT, AST, creatinine, uric acid and urea were measured on an automated clinical biochemistry analyzer (Mindray).

**Lentiviral production**

Lentiviral particles were produced by transfecting HEK293T cells with a pLVX-Puro transfer vector (encoding mouse CD19, chGPC3 or mouse PD-L1) together with second-generation packaging plasmids (psPAX2 and pMD2.G) using Lipofectamine 2000 (11668019, Invitrogen). Supernatants were harvested 48 h after transfection, filtered through a 0.45 µm PES filter (SLHPR33RB, Millipore), and concentrated using Lenti-X concentrator (631231, Takara). Target cells were infected with concentrated virus for 24 h, followed by puromycin selection (4 µg/mL for CT26 and Hepa1-6; 8 µg/mL for CHO-K1) to generate CT26-mCD19, Hepa1-6-chGPC3 and CHO-mPD-L1 stable cell lines.

**Retroviral production**

Murine CAR constructs (αmCD19-CAR and αGPC3-CAR) consisted of a mouse CD8a signal peptide, antigen-specific scFv, CD8a hinge region, mouse CD28 transmembrane and co-stimulatory domain, and CD3ζ intracellular domain, and were cloned into a pMSCV vector. For mCherry labeling, a P2A-mCherry cassette was inserted downstream of the CAR sequence. Retrovirus was produced by transfecting Plat-E cells with pMSCV vectors and pCL-Eco (1:1) using Lipofectamine 2000. Culture medium was collected 48 h later and passed through a 0.45 μm PES filter. The virus was obtained by further concentrating using Lenti-X concentrator. All virus was aliquoted and stored at -80 °C.

**Murine CAR-T cell generation**

Briefly, spleen from BALB/c or B6.SJL-*Ptprc^a^ Pepc^b^*/BoyCrl (B6-CD45.1) mice were harvested and T cells were purified with mouse T cell isolation kit (19851, STEM CELL). Purified mouse CD3 T cells were cultured in RPMI-1640 supplemented with 10% fetal bovine serum, 1% penicillin/streptomycin, 1 mM sodium pyruvate (11360070, Gibco), 50 μM *β*-ME (abs9592, absin), 10 mM HEPES (15630080, Gibco), 1× MEM NEAA (11140050, Gibco), 20 ng/mL recombinant mouse IL-2 (CK24, novoprotein), and Dynabeads Mouse T-Activator CD3/CD28 (at bead-to-cell ratio of 2:1, 11452D, Gibco) for 2 days in a humidified CO_2_ incubator at 37°C. Then, 2 days after activation, T cells were collected, washed and resuspended at 1 × 10^6^ cells/mL in concentrated retroviral supernatant. Spinfection (1,500 g for 90 min at 32 °C) was performed in a 24-well RetroNectin (T100A, Takara)-coated plates at to express a mouse CD19 CAR construct or a GPC3 CAR construct. The activation beads were removed after 24 h. Transduced cells were maintained at 1 × 10^6^ cells/mL in T cell culture medium. To assess CAR expression 48 h after transduction, T cells were stained with mouse CD19 protein (50510-M02H, SinoBiological) or human GPC3 (CW66, novoprotein) protein on ice for 1h, followed by FITC-conjugated Goat anti-human IgG(H+L) (1:20; SA00003-12, proteintech). CAR-T cells were ready for adoptive transfer into experimental models 48 h post-transduction.

***In vitro* CAR-T c****ytotoxicity and proliferation assays**

2 × 10^4^ luciferase-expressing CT26-mCD19 cells, 1 × 10^4^ αmCD19-CAR-T cells, and 1 × 10^3^ dendritic cells were seeded into white opaque 96-well plates. Different treatments were added as required, and the cells were incubated at 37 °C with 5% CO₂ for 24 h. Cells were lysed and luciferase activity was measured using the Luciferase Assay System (E1500, Promega) on a Synergy microplate reader (BioTek). Percent killing was calculated as: 100 – [(RLU_sample_ – RLU_blank_) / (RLU_target only_ – RLU_blank_) × 100].

For proliferation and activation assays, target cells, CFSE-labeled CAR-T cells and BMDCs were seeded in 12-well plates at a ratio of 20:10:1. After 24 h, CD69 expression on CD8^+^ T cells was analyzed by flow cytometry. After 3 days, CFSE dilution was assessed by flow cytometry.

**CAR-T t****rafficking assays (*ex vivo* and *in vivo*)**

For *ex vivo* trafficking assays, mice bearing approximately 150 mm^3^ CT26-mCD19-Luc tumors received PBS, OMVs or OMV^PD-L1 scFv^ (1 mg/kg, i.v.). Tumors were harvested 1 day later and co-cultured with 5 × 10^5^ DiR-labeled αmCD19-CAR-T cells in complete RPMI 1640 supplemented with gentamicin (50 µg/mL; G1264, Sigma) for 24 h. DiR signal was quantified by IVIS (PerkinElmer, USA).

For *in vivo* trafficking assays, mice were injected intravenously with 2 × 10^6^ DiR-labeled αmCD19-CAR-T cells 24 h after receiving PBS or OMV formulations. IVIS imaging was performed every 2 days to quantify DiR signal in the tumor region.

**Antibodies**

Super Bright 600-anti-mouse CD45 (clone: 30-F11) (63-0451-82, Invitrogen), FITC-anti-mouse CD3 (clone: 145-2C11) (11-0031-82, Invitrogen), APC-anti-mouse CD4 (clone: RM4-5) (17-0042-82, Invitrogen), PE-Cy7-anti-mouse CD8α (clone: 53-6.7) (25-0081-82, Invitrogen), PE-Cy5.5-anti-mouse CD25 (clone: PC61.5) (35-0251-82, Invitrogen), and PE-anti-mouse Foxp3 (clone: FJK-16s) (12-5773-82, Invitrogen), APC-anti-mouse CD69 (clone: H1.2F3) (17-0691-82, Invitrogen) , PE-anti-mouse PD-1 (clone: 29F.1A12) (135205, Biolegend), and PE-Cy7-anti-mouse TIM-3 (clone: RMT3-23) (17-0691-82, Invitrogen), PerCP-Cy5.5-anti-mouse CD8 (clone: 53-6.7) (45-0081-80, Invitrogen), BV605-anti-mouse Ki67 (clone: B56) (567122, BD Biosciences), APC-Cy7-anti-human/mouse Granzyme B (clone:QA16A02) (372228, Biolegend), eFluor 450-anti-mouse F4/80 (clone: BM8) (48-4801-82, Invitrogen), PE-eFluor 610-anti-mouse CD11b (clone: M1/70) (61-0112-82, Invitrogen), FITC-anti-mouse CD11c (clone: N418) (11-0114-82, Invitrogen), PE-anti-mouse MHCII (clone: NIMR-4) (clone: 30-F11), V500-anti-mouse CD45.2 (clone: 104) (562130, BD Biosciences), RY610-anti-mouse CD45.1 (clone: A20) (759388, BD Biosciences), PE-Cy7-anti-mouse CD86 (clone: GL1) (25-0862-80, Invitrogen), PerCP-eFluor 710-anti-mouse CD206 (clone: MMR) (46-2061-82, Invitrogen), APC-anti-mouse CD80 (clone: 16-10A1) (25-0862-80, Invitrogen), BV421-anti-mouse CCR7 (clone: 4B12) (566291, BD Biosciences), APC-anti-mouse CD44 (clone: IM7) (17-0441-82, Invitrogen), PE-anti-mouse CD62L (clone: MEL-14) (12-0621-81, Invitrogen), PE-Cy7-anti-mouse IFN-γ (clone: XMG1.2) (25-7311-82, Invitrogen), eFluor450-anti-mouse CD4 (clone: GK1.5) (48-0041-80, Invitrogen), BB700-anti-mouse CD103 (clone: 2E7) (748240, BD Biosciences) and RY703-anti-mouse F4/80 (clone: T45-2342) (770168, BD Biosciences). Live/Dead staining using Fixable Viability Dye eFluo 450 (65-863-14, Invitrogen), Fixable Viability Dye eFluo 506 (65-0866-18, Invitrogen), or Fixable Scarlet (723) (L34986, Invitrogen).

**Single-cell library preparation and sequencing**

C57BL/6 mice were inoculated (s.c.) with Hepa1-6-chGPC3 cells and received (i.v.) αGPC3-CAR-T treatment and OMV^PD-L1 scFv^ combined αGPC3-CAR-T. The intratumoral CD45^+^ cells were isolated by flow cytometry for scRNA-seq on day 5 post CAR-T transfer.

In brief, tumor-infiltrating CD45^+^ cells were isolated from each group using the EasySep™ Mouse CD45 Positive Selection Kit (100-0350, STEMCELL Technologies) and quantified with a TC20 Automated Cell Counter (Bio-Rad). The cell suspension was loaded onto Chromium GEM-X microfluidic chips to generate single-cell gel beads-in-emulsion (GEMs) using the Chromium Next GEM Single Cell 3' Reagent Kits v4 (10× Genomics). Within the GEMs, lysis occurred and poly-adenylated mRNA was reverse-transcribed into full-length cDNA containing an Illumina Read 1 sequencing primer, a 16 nt 10× Barcode, and a 12 nt unique molecular identifier (UMI). The amplified cDNA was quantified by a Qubit 3.0 Fluorometer (Invitrogen, USA) and Agilent 2100 Bioanalyzer (Agilent Technologies, USA). For library construction, the cDNA was fragmented, end-repaired, and A-tailed, followed by double-sided size selection to recover fragments of ~300 bp. The final libraries were validated using a Qsep100 (Bioptic, Taiwan, China) and quantified by Qubit 3.0 Fluorometer. Sequencing was performed on an Illumina NovaSeq instrument using a 2x150 bp paired-end (PE) configuration. Data processing, including cell calling and transcript counting, was performed using the Cell Ranger (v9.0.1) pipeline.

**Supplementary Figure**

**Supplementary Figure S1.** **Design of the main** **plasmid constructs.**

**Supplementary Figure S2. Expression and targeting validation of** **OMV^Her2-scFv^. (A)** Immunoblot analysis of ClyA–Her2-scFv–6×His expression in purified vesicles, detected with anti-His antibody. **(B)** Representative fluorescence images of 4T1 and 4T1-hHer2 cells for OMV^Her2-scFv^ uptake. Scale bars: 200 µm.

**Supplementary Figure S3. *Ex vivo* IVIS imaging of tumors 12 and 48 hours after intravenous injection of DiR-labeled OMVs or** **OMV^PD-L1 scFv^.**

**Supplementary Figure S4. OMV formulations promote BMDC maturation.** Flow cytometry quantification of MHC-II^+^CD86^+^ cells among CD11c^+^ BMDCs after incubation with PBS, soluble PD-L1 scFv, parental OMVs, OMVs plus scFv, or OMV^PD-L1 scFv^ (n = 3 independent experiments). Data are represented as mean ± SEM. Statistical significance was calculated by one-way ANOVA with a Tukey post-hoc test. ***P < 0.001; ns, not significant.

**Supplementary Figure S5. Binding of recombinant CD28–Fc to lymph node cells from mice treated with different OMVs measured by flow cytometry.** Representative histograms (left) and mean fluorescence intensity (right) (n= 3 mice per group). Data are mean ± SEM. One-way ANOVA with Tukey’s multiple comparisons test. *P < 0.05, **P < 0.01, ***P < 0.001.

**Supplementary Figure S6. OMV^PD-L1 scFv^ shifts M2 macrophage transcriptional programs.** qPCR analysis of *Nos2* (iNOS) (**A**) and *Arg1* (Arg-1) (**B**) in IL-4-polarized M2 BMDMs after treatment with PBS, PD-L1 scFv, OMVs, OMVs plus scFv, or OMV^PD-L1 scFv^ (n = 3 independent experiments). Data are mean ± SEM. One-way ANOVA with Tukey’s multiple comparisons test. **P < 0.01, ***P < 0.001; ns, not significant.

**Supplementary Figure S7.** **Flow cytometric** **gating strategy of T cell infiltration into A20 tumors.**

**Supplementary Figure S8. Flow cytometric gating strategy of DC cell infiltration into A20 tumors.**

**Supplementary Figure S9.** **Histopathology after OMV treatment in the A20 model.** Representative H&E staining of A20 tumors (**A**) and major organs (**B**) collected on day 25 after the indicated treatments. Scale bars: 50 µm (A) and 100 µm (B).

**Supplementary Figure S10.** **OMV^PD-L1 scFv^ inhibits tumor growth** **in the CT26 CRC model.** (**A**) Treatment schematic for the syngeneic CT26 subcutaneous CRC model in BALB/c mice (3 intravenous doses on days 8, 10 and 12 after tumors reached ~100 mm^3^). (B, C) Mean tumor volume growth curves (**B**) and individual tumor growth curves (**C**) (n = 6 mice per group). CR, complete remission. Data are mean ± SEM. (**D**) Representative H&E staining of spleens collected on day 19 from the indicated OMV-treated groups; arrows indicate extramedullary hematopoiesis. Scale bar: 100 µm.

**Supplementary Figure S11.** **Safety evaluation of single-dose OMV^PD-L1 scFv^ administration.** (**A**) Spleen images on days 3 and 7 after a single intravenous dose of OMV^PD-L1 scFv^ compared with negative control (NC). (**B**) Spleen weight (n = 5 mice per group). (**C**) Body weight (n = 4 mice per group). Data are mean ± SEM. (**D**) Representative H&E staining of major organs on days 3 and 7; arrows indicate transient splenic EMH. Scale bars: 100 µm. (**E**) Hematological and serum biochemistry parameters measured at the indicated time points after dosing (n = 5 mice per group). Boxes show median and interquartile range; whiskers denote min/max.

**Supplementary Figure S12.** **Serum concentrations of pro-inflammatory cytokines 12 h, 1 day, 3 days and 7 days post OMV^PD-L1 scFv^ intravenous injection.** NC: normal control. Data are presented as mean ± SEM (n = 4 mice per group).

**Supplementary Figure S13.** **Representative CAR transduction by flow cytometry on αmCD19-CAR-T (A) and αGPC3-CAR-T (B) transduced T cells after staining with mouse CD19 and human GPC3 protein.**

**Supplementary Figure S14.** **Serum biochemistry in the advanced-stage CT26-mCD19 model.** Serum chemistry at the study endpoint (ALP, ALT, AST, creatinine and uric acid) for the indicated treatment groups (n = 4 mice per group). Boxes show median and interquartile range; whiskers denote min/max.

**Supplementary Figure S15.** **OMV^PD-L1 scFv^ combined with CAR-T therapy establishes long-term immunological memory against tumors. (A)** Schematic illustration of the tumor re-challenge experiment. **(B)** Average tumor growth curves of naive mice and cured mice following re-challenge (n=5 for naïve mice; n=3 for cured mice). Data are mean ± SEM. **(C)** Individual tumor growth curves.

**Supplementary Figure S16.** **Flow cytometric** **gating strategy of CAR-T cell and myeloid cells infiltration into Hepa1-6-chGPC3 tumors.**

**Supplementary Figure S17. Flow cytometric gating strategy of mature DCs and tumor-specific T cell in lymph nodes from mice bearing Hepa1-6-chGPC3 tumors.**

**Supplementary Figure S18.** **Dendritic cell maturation and** **tumor-specific T cell responses in lymph nodes under** **OMV^PD-L1 scFv^ pre-treatment.** (A–C) Flow cytometry quantification of the overall percentage of DCs **(A)**, mature DCs **(B)**, and mature migratory CD103^+^ CCR7^+^ DCs **(C)** in the non-draining lymph nodes (ndLN) and tumor-draining lymph nodes (tdLN) of mice treated with CAR-T alone or OMV^PD-L1 scFv^ + CAR-T. **(D)** Representative flow cytometry plots illustrating the expression of maturation markers (CD80^+^ CD86^+^) on DCs in the tdLN and ndLN. (E, F) Quantitative analysis of the percentage of intracellular IFN-γ-producing cells within the CD4^+^ T cell **(E)** and CD8^+^ T cell **(F)** compartments in the ndLN and tdLN. **(G)** Representative flow cytometry plots of IFN-γ expression in CD4^+^ and CD8^+^ T cells. **(H)** Statistical quantification of effector memory (Tem) and central memory (Tcm) T cell subsets within the CD4^+^ and CD8^+^ populations in the tdLN (top) and ndLN (bottom). **(I)** Representative flow cytometry plots for identifying T cell memory subsets based on CD62L and CD44 expression (Naive: CD62L^+^ CD44^-^; CM: central memory, CD62L^+^ CD44^+^; EM: effector memory, CD62L^-^ CD44^+^). Data are presented as mean ± SEM. Statistical significance was calculated using Student's t-test (A-F, H). *P < 0.05, **P < 0.01, ***P < 0.001; ns, not significant.

**Supplementary Figure S19.** **Cell-type annotation, pathway enrichment and expanded CellChat analyses.** (**A**) Feature plots visualizing the expression distribution of canonical marker genes for distinct cell clusters on the UMAP projection. Darker red indicates higher expression levels. (**B**) Heatmap of signature genes in 9 different clusters of immune cells. The signature genes are listed on the x axis. (**C**) Gene Ontology (GO) enrichment analysis of differentially expressed genes (DEGs) between CAR-T and OMV^PD-L1 scFv^ + CAR-T groups. (**D**) Kyoto Encyclopedia of Genes and Genomes (KEGG) pathway enrichment analysis of DEGs. (**E**) Heatmap of the differential number of interactions between defined cell subsets. Red and blue squares indicate increased and decreased interaction numbers in the OMV^PD-L1 scFv^ + CAR-T compared to the CAR-T group, respectively. Adjusted P values for Gene Ontology and KEGG pathway enrichment analyses were calculated using the Benjamini–Hochberg method to control the false discovery rate (FDR).

**Supplementary Figure S20.** **Deep profiling of T cell transcriptional states.** (**A**) Quantile–quantile (Q–Q) plots (top panels) and corresponding UMAP feature plots (bottom panels) for representative genes linked to dendritic cell chemotaxis (*Xcl1*), NK/T cell activation (*Cd69*, *Klrk1*), cytotoxicity (*Gzma*, *Gzmb*, and *Prf1*), memory/stemness phenotypes (*Bcl2*, *Eomes*, and *Tcf7*) and exhaustion (*Lag3*, *Tigit*, and *Havcr2*) between OMV^PD-L1 scFv^ + CAR-T and CAR-T groups. (**B**) UMAP density distribution of proliferation markers *Mki67* (left) and *Top2a* (right) across immune and tumor area. (**C**) Dot plot visualizing the gene expression landscape of T cell subclusters. Rows represent defined T cell subsets, and columns display lineage markers (*Cd4*, *Cd8a*) alongside activation/effector genes, co-stimulation/co-inhibition gene, differentiation/memory/homing genes and transcription-factor genes.

**Supplementary Figure S21.** **DC and macrophage subcluster and marker validation.** (**A**) UMAP of re-clustered dendritic cells (six subtypes). (**B**) Relative proportions of DC subtypes across groups. (**C**) Dot plot of canonical marker genes across DC subclusters. (**D**) Bubble plot of CD86–CD28 and CD80–CD28 co-stimulatory pairs from DC subsets (including moDCs and pDCs) to CD4^+^ and CD8^+^ T cells (expanded view). (**E**) Integrated UMAP of re-clustered macrophages (four subtypes). (**F**) Split UMAP showing macrophage subclusters by treatment group. (**G**) Dot plot of canonical marker genes across macrophage subclusters. (**H**) Analysis of metabolic module scores in macrophages. Violin plots quantifying the expression scores for Glycolysis (left) and Oxidative Phosphorylation (OXPHOS) (right). (**I**) Correlation between macrophage glycolysis and OXPHOS module scores. (Glycolysis gene sets: *Hk2*, *Ldha*, *Gapdh*, *Pkm*, *Slc2a1*; OXPHOS gene sets: *Atp5a1*, *Cox5a*, *Ndufb8*, *Sdha*, *Uqcrc2*). Statistical significance was determined using a two-sided Wilcoxon rank-sum test. *P < 0.05, **P < 0.01, ***P < 0.001; ns, not significant.
